# Population Pharmacokinetic (Pop-PK) Analysis of Torsemide in Healthy Korean Males Considering CYP2C9 and OATP1B1 Genetic Polymorphisms

**DOI:** 10.3390/pharmaceutics14040771

**Published:** 2022-04-01

**Authors:** Seung-Hyun Jeong, Ji-Hun Jang, Hea-Young Cho, Yong-Bok Lee

**Affiliations:** 1 College of Pharmacy, Chonnam National University, 77 Yongbong-ro, Buk-gu, Gwangju 61186, Korea; rhdqn95@naver.com (S.-H.J.); jangji0121@naver.com (J.-H.J.); 2 Department of Pharmaceutical Sciences, School of Pharmacy and Pharmaceutical Sciences, State University of New York at Buffalo, Buffalo, NY 14214, USA; 3 College of Pharmacy, CHA University, 335 Pangyo-ro, Bundang-gu, Seongnam-si 13488, Gyeonggi-Do, Korea

**Keywords:** torsemide, population pharmacokinetic modeling (Pop-PK), CYP2C9, OATP1B1, inter-individual variability (IIV)

## Abstract

Torsemide is a diuretic drug used for several cardiovascular and chronic diseases. With regard to the clinical application of torsemide, studies on individualized pharmacotherapy and modeling that take variability in pharmacokinetics (PKs) within a population into account have been rarely reported. Thus, the objective of this study was to perform population pharmacokinetic (Pop-PK) modeling and to identify effective covariates that could explain the inter-individual variability (IIV) of torsemide PK. Pop-PK modeling for torsemide was performed based on serum concentration data obtained from 112 healthy Korean males and analysis of various genetic and physicochemical parameters. Modeling was performed with nonlinear mixed-effects (NLME) using Phoenix NLME. The finally developed model was fully verified. The model was also reconfirmed using NONMEM software. As a basic model, the PKs of torsemide within the population were well described by a two-compartment model reflecting the lag-time on oral absorption. According to the genetic polymorphisms of OATP1B1 and CYP2C9, significant associations were found in the V/F, CL/F, and CL_2_/F of torsemide. These were reflected as effective covariates in the final Pop-PK model of torsemide, resulting in an approximately 5–10% improvement in the model parameter IIV values. Considering that torsemide is a substrate for CYP2C9 and OATP1B1, it was important to search for genetic polymorphisms in CYP2C9 and OATP1B1 as covariates to explain the PK diversity of torsemide between individuals. The differences in CL/F and CL_2_/F between the phenotypes of CYP2C9 were approximately 36.5–51%. The difference in V/F between the phenotypes of OATP1B1 was approximately 41–64.6%. These results suggested that the phenotypes of CYP2C9 and OATP1B1 produced significant differences in torsemide PKs. Considering that CYP2C9 and OATP1B1 phenotypes as covariates affected different PK parameters of torsemide, it could be inferred that torsemide’s cell membrane permeation process by OATP1B1 and the metabolic process by CYP2C9 could independently affect each other in vivo without interplay. There was no significant difference in the parameter estimates between modeling software (Phoenix NLME vs. NONMEM). In this study, the torsemide PK variability between individuals was largely explained. In the future, individualized effective drug therapy of torsemide taking individual patient’s genotypes into account might become possible.

## 1. Introduction

Torsemide (1-isopropyl-3-(4-[3-methyl-phenyl-amino]pyridine)-3-sulfonylurea) is also a drug called torasemide [1]. Structurally, it is a loop diuretic drug belonging to the sulfonamide class. Its site of action is the thick ascending limb of Henle’s loop [2]. Its mechanism of action is that it can greatly increase the excretion of Na^+^, K^+^, and Cl^−^ in the urine by inhibiting the Na^+^/K^+^/2Cl^−^ symporter [2]. As a result, the osmotic gradient for water reabsorption is reduced with a diuretic effect. In the clinic, torsemide is used to help treat fluid retention (edema) and swelling caused by congestive heart failure, liver disease, and kidney disease [3]. Common side effects of torsemide include headache, increased urination, diarrhea, cough, and fatigue. Side effects of hearing loss and hypokalemia have also been reported [4].

In clinical practice, torsemide is administered orally or intravenously [5]. According to past reports [1,5], the bioavailability of torsemide was as high as 80–90%, indicating that torsemide could be well absorbed through the gastrointestinal tract into the bloodstream. It has been reported that the protein binding degree of torsemide is very high, at over 99% [1,6]. The in vivo metabolism of torsemide mainly occurs in the liver. It has been reported that most metabolism is through an oxidative reaction by CYP [7]. Its main metabolites have various oxidative forms, such as hydroxylation of the methyl group of the phenyl ring, carboxylic acid forms due to additional oxidation, and a hydroxylation form of the phenyl ring [1,8]. Therefore, it could be judged that the difference in activity according to the genetic polymorphism of CYP will have a major effect on the metabolism of torsemide in the body. Previous reports [8,9] have confirmed that torsemide is a substrate of CYP2C9 and that the metabolism of torsemide differs depending on the polymorphism of CYP2C9. For example, it has been reported that the metabolism of torsemide in the body decreases as the genotype of CYP2C9 goes from *1*1 to *1*3 and *3*3. In addition, as a result of confirming the relationship between the polymorphisms of various enzymes in the CYP2C family (such as CYP2C8, CYP2C9, CYP2C18, and CYP2C19) and the degree of torsemide metabolism, there was a high negative association between the number of CYP2C9*3 alleles and the degree of torsemide metabolism [7,9]. The excretion of torsemide metabolites (including some unchanged torsemide) has been reported to occur primarily through urine [1]m, and within-group variability in torsemide renal clearance has been reported to be associated with genetic polymorphisms in OAT4 [10]. However, the degree of renal function is not related to the in vivo clearance of torsemide [1,11]. This is probably because the clearance of torsemide through the kidneys is not high (about 20% of the total clearance of torsemide in the body) [1]. Because of the extensive metabolism of torsemide in the liver, the extent of renal function has little effect on the clearance of torsemide [11].

Previous in vitro cellular uptake studies [12,13,14] have confirmed that torsemide is a substrate of OATP1B1. A difference in the degree of cellular uptake of torsemide according to the genetic mutation of OATP1B1 has been confirmed by an in vitro experiment [14]. OATP1B1 is a tissue-specific transporter mainly expressed in hepatocytes [15]. OATP1B1 is located in the basolateral membrane of hepatocytes [15]. Although the in vivo pharmacokinetic (PK) diversity degree of torsemide according to OATP1B1 polymorphism has not been clearly identified yet, the genetic polymorphism of OATP1B1 might be related to the interindividual PK diversity of torsemide. That is, considering that torsemide is a substrate of OATP1B1 and that OATP1B1 is expressed on the basolateral side of hepatocytes, there might be significant associations between the distribution of torsemide in the body and the genetic polymorphisms of OATP1B1. A previous study [14] reported that the PK diversity of torsemide within a group of patients with cardiovascular disease is large, which could be explained by the genetic polymorphism of OATP1B1.

The main purpose of this study was to discover effective covariates that could quantitatively explain the degree of diversity in the PK diversity of torsemide within the population, and to perform mathematical modeling reflecting these covariates. The importance of torsemide in clinical practice and the scope of its application are very large. This is because diuretics are widely prescribed for cardiovascular and other chronic diseases. In addition, torsemide has the advantage of having a relatively higher effect than other diuretic drugs such as furosemide [1]. Therefore, studies on the PK of torsemide, especially clinical studies on inter-individual variability (IIV), are very important to make it possible to develop an optimal clinical drug therapy taking into account the PK variability of torsemide among individuals. That is, the practical application of personalized drug therapy that can minimize the side effects of torsemide and maximize the effect will gradually become possible through PK diversity studies like this one, based on healthy subjects further to patients [16,17,18,19]. By first clarifying the covariates associated with PK fluctuations in healthy adults, it will be possible to determine the extent of covariate effects in patient populations later.

Most clinical studies of torsemide reported in the past [20,21,22,23,24,25,26,27,28,29] mainly presented and compared the values of some PK parameters in the test group through non-compartment analysis (NCA). There have been few reports of population PK (Pop-PK) modeling studies considering the intra- or inter-individual PK variability of torsemide. Simply presenting and comparing PK parameters through NCA could not clearly explain the PK variability or related key factors with objective results. Through Pop-PK modeling, we can discover significant covariates that can explain the PK variability within a group and estimate the extent of influence by the covariates. As mentioned earlier, if factors related to PK fluctuations in healthy adults are identified first, it will be possible to find out the magnitude of change in PK fluctuation factors in patients based on the results obtained from healthy adults. As a result, it will be possible to know to what extent the disease affects the magnitude of change in PK fluctuation factors, which will help the disease management and treatment of patients.

Although Pop-PK modeling of torsemide considering genetic polymorphisms of CYP2C9 has been attempted in the past [8], the results for model development were not satisfactory. That is, the process of verification and the presentation of the results related to the Pop-PK model were not performed at all. This is probably because the identification of PK diversity according to the genotype difference of CYP2C9 was the main objective, rather than the reporting of the modeling in the previous study [8]. In addition, modeling was attempted with limited data obtained after a single oral administration of 10 mg of torsemide to 36 healthy adults. Therefore, the need for additional studies using data from more individuals has been raised in the previous report [8].

Based on PK results obtained from a large number of healthy Korean men in this study, we performed Pop-PK modeling of torsemide, which had not been reported previously. This study aimed to explore the genetic factors that explain the PK variability of torsemide within the population and to objectively analyze the effects of these factors on each PK parameter. The Pop-PK modeling results for torsemide presented from this study will be useful for understanding the PK properties of torsemide in humans more clearly than before. It is expected that this study will serve as a scientific reference for determining the dose regimen of torsemide in the clinic.

## 2. Materials and Methods

### 2.1. Materials

Torsemide and furosemide standards were purchased from Sigma-Aldrich (St. Louis, MO, USA). The chemical structures of torsemide and furosemide are presented in Figure S1. Methanol, acetonitrile, and ethyl acetate were purchased from Fisher Scientific (Hampton, NH, USA). All had high-performance liquid chromatography (HPLC) grade purity. A Milli-Q water purification system (Millipore Co., Burlington, MA, USA) was used to obtain purified water for the HPLC.

### 2.2. Subjects

A total of 112 healthy Korean males (derived from a total of four bioequivalence tests) were included in the Pop-PK model study for torsemide. Women were not included in these bioequivalence tests. All subjects were enrolled in studies evaluating the bioequivalence between conventional marketed torsemide and generic drugs. In this study, only samples obtained after administration of the standard reference formulation were used for analysis and Pop-PK modeling, and the reference formulation of torsemide was identical in all four bioequivalence tests. In the questionnaire conducted prior to the clinical trials, none of the subjects had had a hypersensitivity reaction to sulfonamide drugs in the past. The subjects had no history of congenital or chronic diseases, either. They were all physically normal. Prior to participation in the bioequivalence and PK study, all subjects provided written informed consent. All subjects underwent a comprehensive physical examination, blood test, clinical chemistry test, and urine test just before participating in the torsemide clinical study. Only subjects whose test results were within normal ranges were selected as final test subjects. Additionally, the subjects were requested to refrain from taking other medication or alcohol for at least one week prior to this study. The Institutional Review Board (IRB) of the Institute of Bioequivalence and Bridging Study, Chonnam National University (Gwangju, Republic of Korea), approved the study protocol (approval numbers: 710; 29 May 2004, 805; 2 June 2005, 330; 10 February 2006, and 521; 11 April 2007). These studies followed the revised Declaration of Helsinki for Biomedical Research with human subjects and the Rules of Good Clinical Practice. Each bioequivalence study was conducted using an open-label single dose, randomized, two-period crossover design with a one-week washout.

### 2.3. Sampling

A heparin-locked (150 unit/mL) angiocatheter (JELCO 22G, Smiths Medical, Minneapolis, Minneapolis, MN, USA) was installed into the veins of subjects’ arms or hands for adequate sampling prior to torsemide administration. About 8 mL was collected as blank blood sample before administration (0 h). All subjects were then given a single dose of torsemide tablets orally with 240 mL of water. Torsemide doses at 5, 10, and 20 mg were given to 28, 28, and 56 subjects, respectively. Blood samples were taken from all subjects at 0.5, 1, 1.5, 2, 3, 4, 5, 6, 8, 10, and 12 h post-dose. Prior to each sampling, 2 mL of blood was first discarded (to completely remove heparinized saline solution remaining in the venous catheter during blood collection) and then approximately 8 mL of blood was collected and placed in Vacutainer tubes (BD, Franklin Lakes, NJ, USA). Saline for injection containing heparin was injected into the catheter after each blood collection to prevent the clotting of the blood remaining in the venous catheter. Blood samples were centrifuged (20 min and 3000× *g*) and then the supernatant was transferred to serum separation tubes and stored at −80 °C until analysis.

### 2.4. Determination of Clinical Biochemistry Parameters

Blank serum samples obtained immediately before oral torsemide administration (as 0 h) were used for the analysis of clinical biochemical parameters. The analysis of the biochemical parameters was only performed for the 20 mg dose group (*n* = 56). The types of biochemical parameters included albumin, total proteins, total bilirubin, aspartate transaminase (AST), alkaline phosphatase (ALP), alanine transaminase (ALT), cholesterol, creatinine, blood urea nitrogen (BUN), glomerular filtration rate (GFR), and creatinine clearance (CrCl). The determination of clinical biochemical parameter values was performed by serological analysis through a dry automated analyzer of microsides VITROS (Ortho Clinical Diagnostics, NJ, USA) operating with a reflectance spectrophotometry.

### 2.5. Determination of Genotypes

In this study, CYP2C9 and OATP1B1 were selected as potential genetic target covariates that could explain the PK diversity of torsemide within a population. The genotyping of CYP2C9 and OATP1B1 was performed for all subjects (*n* = 112) in the clinical trials.

#### 2.5.1. Identification of CYP2C9*3 and *13 Alleles

Genotypes of CYP2C9*3 and *13 alleles for each individual were determined by referring to the previously reported polymerase chain reaction–restriction fragment length polymorphism (PCR–RFLP) method [16,30]. Detailed conditions and procedures for PCR–RFLP involved in the determination of CYP2C9 alleles are presented in the Supplementary Information (*Methods for determining genotypes* section).

#### 2.5.2. Identification of SLCO1B1 388A>G and 521T>C

Genetic information about OATP1B1 is encoded in the SLCO1B1 gene [15]. The genotypes of SLCO1B1 388A>G and 521T>C were determined for each individual using PCR–RFLP methods with reference to a previous report [31]. Detailed conditions and procedures for PCR–RFLP involved in the determination of SLCO1B1 genotypes are presented in the Supplementary Information (*Methods for determining genotypes* section).

### 2.6. Determination of Serum Torsemide Concentration

The torsemide concentrations in the serum samples from all subjects were determined using the high-performance liquid chromatography-ultraviolet (HPLC-UV) assay established and validated in previous studies [20,21]. The verification items included selectivity and specificity of analysis, linearity, precision, accuracy, carryover, recovery, stability, and incurred sample reanalysis. All assay validations were performed in accordance with FDA’s Bioanalytical Method Validation guidelines [32]. Detailed analysis conditions (including chromatographic conditions, calibration curve, and sample preparation) related to the determination of torsemide serum concentrations are presented in the Supplementary Information (*Assay conditions to determine serum torsemide concentration* section).

### 2.7. PK Analysis

Phoenix WinNonlin software version 8.3 (Pharsight, Certara Inc., Princeton, NJ, USA) was used for the calculation of the basic PK parameters of torsemide via NCA. The area under the curve from 0 h to infinity (AUC_0–∞_) was calculated as the sum of AUC_0–t_ and C_last_/k, where C_last_ was the final measurable concentration, t was the time in C_last_, and k was the elimination rate constant at the terminal phase. k was calculated by obtaining a regression line for more than 3–4 points in the elimination phase. The adjusted correlation coefficient (R^2^) value of the regression line was 0.8 or higher as an acceptable standard. AUC_0–t_ from 0 to t h after oral administration of torsemide was calculated using the linear trapezoidal rule. The degree of extrapolated AUC (as AUC_t–∞_) was less than 20% as an acceptable criterion. The half-life (T_1/2_) was calculated as 0.693/k and the volume of the distribution (V/F) was calculated as dose/k · AUC_0–∞_. The clearance (CL/F) was calculated by dividing the dose of torsemide by AUC_0–∞_, where F meant the bioavailability of oral administration. The quantitative results obtained using HPLC-UV were plotted as torsemide serum concentration (*Y*-axis) versus time (*X*-axis), and the highest drug concentration in serum (C_max_) and time to reach C_max_ (T_max_) were determined from each individual’s serum torsemide concentration–time curve. All PK parameter values (by NCA) were estimated as mean ± standard deviation (SD). The statistical significance of differences between groups according to the genetic polymorphisms in the PK parameter values was confirmed using a Student’s *t*-test or one-way analysis of variance (ANOVA). Statistical significance was considered when the *p*-value was less than 0.05.

### 2.8. Model Development

The Pop-PK model of torsemide was developed using the non-linear mixed effects (NLME) model approach and first-order conditional estimates (FOCE) method. This method was an extended least squares estimate with *ŋ*–*ε* interaction. The overall Pop-PK model analysis of torsemide was performed using Phoenix NLME software version 8.3 (Pharsight, Certara Inc.). Torsemide’s Pop-PK model analysis and construction were largely performed with the following three steps. In the first step, a basic structural model that could explain the PKs of torsemide relatively well in the population was established. In the second step, significant covariates that could explain the PK variability of torsemide between individuals were identified. In the third step, a model that could explain the experimentally obtained Pop-PK results of torsemide as a whole was finally established by applying covariates obtained in the second step to the basic structural model established in the first step.

#### 2.8.1. Establishment of Structural Base Model

To establish a compartment model suitable as a basic model structure, serum torsemide concentration data over time from 112 patients were applied to various compartment models. Each tried compartment model differed in the number of disposition compartments (such as 1, 2, or 3), the drug movement rate between compartments (such as zero- or first-order), and whether the lag-time was reflected in the oral absorption of torsemide (such as with or without lag-time). The final selection of the structural base model was performed by comparing differences of statistical significance, such as twice the negative log likelihood (−2LL) and Akaike’s information criterion (AIC) values, between the models. In this process, changes in statistical significance (based on *Chi-square distribution*) with an increasing or decreasing number of parameters were also considered. In the final selection of the structural base model, the degree of visual agreement related to the model fitting of the data was considered by comparing goodness-of-fit (GOF) plots between the models. Proportional, additive, log-additive, mixed, and power error models were tried as models to account for residual variability. The IIV in the PK parameters of torsemide was evaluated using an exponential error model with the assumption that the parameters followed a log-normal distribution. The exponential error model had the following formula: P_i_ = P_tv_ · exp(*ŋ*_i_), where *ŋ*_i_ meant the random variable for the ith individual, normally distributed with mean 0 and variance ω^2^. P_i_ meant the parameter value of the ith individual, and P_tv_ meant a typical value of the population parameter. The exponential error model had the advantage of being able to estimate positive PK parameter values. Finally, it was confirmed whether the consideration of IIV in each PK parameter constituting the structural base model had a major impact on model improvement. That is, whether −2LL and AIC values fluctuated to a significant level along with the reduction in the number of total parameters by removing the IIV consideration became the judgment indicator of model improvement.

#### 2.8.2. Covariate Analysis

Physicochemical and genetic polymorphism data were applied as potential covariates that could explain the PK diversity of torsemide among individuals. Physicochemical information was used as continuous data, including physiological information (such as age, height, and body weight), parameter values calculated based on physiological information (such as body mass index (BMI) and body surface area (BSA)), and biochemical parameters. The degree of correlation between the PK parameters calculated by NCA and the physicochemical parameters for each individual was first screened through regression analysis. Possible candidate covariates were sequentially tried in the IIV model. Genetic polymorphism information was sequentially applied to the IIV model of PK parameters constituting the model as categorical data. Regarding the explanation of IIV, this was confirmed by applying exponential, power, and additive options sequentially as a suitable model in the process of reflecting each covariate. In order to determine the reflection fit of the covariates in the final Pop-PK model, the stepwise forward addition and backward removal of each covariate were performed on the basic structural model. Whether to include covariates in the model was determined based on the degree of change in the objective function value (OFV). Covariates with a decrease in OFV values greater than 3.84 (*p* < 0.05, with an increase in the number of parameters by 1) were included in the base model according to the forward addition procedure. Through the backward elimination process, covariates with an increase in OFV values greater than 6.63 (*p* < 0.01, with a decrease in the number of parameters by 1) were not removed from the model, but remained in the model.

### 2.9. Model Evaluation

The evaluation and validation of the finally established torsemide Pop-PK model was performed both visually and numerically. Phoenix NLME and R software (R Core Team) were utilized to perform all processes of the model evaluation and validation. GOF (with residual distribution), visual predictive check (VPC), bootstrapping, normalized prediction distribution error (NPDE), and external validation were utilized as tools for model evaluation. GOF was identified using several diagnostic scatterplots, and the relationships between the scatterplots were as follows: observed (DV) versus population-predicted (PRED) concentrations, DV versus individual predicted (IPRED) concentrations, conditional weighted residuals (CWRES) versus PRED, CWRES versus time (IVAR), and CWRES versus quantile–quantile plot of the components. The VPC option of Phoenix NLME was used to run the VPC of the finally built model, and the total number of simulations applied was 1000. DV-IVAR data were graphically superimposed on the median, 95th, and 5th percentiles of the simulated concentration–IVAR profile. The visual model accuracy was judged by whether the DV data were roughly distributed within the 95th and 5th prediction intervals. Nonparametric bootstrap analysis was applied to evaluate the stability of the final model, for which the iterative bootstrap option of Phoenix NLME was used. As a result, a total of 1000 replicates were generated, which was achieved through repeated random sampling from the original data set. After bootstrapping, the median and confidence intervals (CIs) of the estimated parameters were compared with the estimated values (with standard errors (SE)) in the original dataset. NPDE analysis was performed to evaluate the predictive performance of the model. This was done based on Monte-Carlo simulations using the R package. The NPDE results were summarized graphically using (A) a quantile–quantile plot of the NPDE, (B) a histogram of the NPDE, (C) a scatterplot of IVAR versus NPDE, and (D) a scatterplot of PRED versus NPDE. If the predictive performance was satisfied, the NPDE would follow a normal distribution (Shapiro–Wilk test) with a mean value of zero (*t*-test) and a variance of one (Fisher’s test). External validation was additionally performed using the VPC of the torsemide Pop-PK model finally established in this study. As an external validation data set, plasma concentration values obtained after the oral administration of torsemide 10 mg reported in a previous study [8] were used. The data were derived from 34 healthy adults and numerically digitized using WebPlotDigitizer (4.5 version). External validation was performed by superimposing digitized data to the VPC of the finally established torsemide Pop-PK model to calculate the number of overlapped observations. The larger the number of observations included in the VPC region, the better the external validation results.

### 2.10. Model Reconfirmation

The torsemide Pop-PK model was further verified using another NLME software, NONMEM (7.3 version, ICON plc, Dublin, Ireland) to check how much the PK parameter values of torsemide in the population estimated using the Phoenix NLME software differed from those calculated with other modeling software. That is, to reconfirm the reliability and accuracy of the torsemide Pop-PK model, the model operation and parameter estimations were performed using additional modeling software. For the NONMEM procedures, ADVAN4 and TRANS4 subroutines from the PREDPP library were used. As an additional parameter, ALAG1 representing the absorption delay time of the storage compartment was applied. Estimation of the parameters was performed with the FOCE method. Other options (such as residual error and IIV) related to the model structure setting were applied in the same way as in Phoenix NLME.

## 3. Results and Discussion

### 3.1. Study Design and Demographic Analysis

For the analysis of the Pop-PK model of torsemide, the results of the bioequivalence tests performed for 112 healthy Korean men were used retrospectively. The mean age, weight, and height of these 112 participants in the bioequivalence tests were 23.38 years (range, 19–29 years), 67.84 kg (range, 44–88.4 kg), and 173.78 cm (range, 159.9–188.1 cm), respectively. The results of the physical examination, hematology, clinical chemistry, and urine tests of all subjects participating in the clinical trials were within normal ranges. In the additional clinical biochemical parameter tests performed after the oral administration of 20 mg torsemide, the mean values of albumin, total protein, creatinine, and CrCl were 4.86 g/dL (range, 4.4–6.6 g/dL), 7.39 g/dL (range, 6.6–8.3 g/dL), 0.94 mg/dL (range, 0.8–1.1), and 117.23 mL/min (range, 78.77–176.39), respectively. The mean values of BMI and BSA were 22.41 kg/m^2^ and 1.81 m^2^, respectively. The mean value of CrCl normalized with BSA was 64.67 mL/min/m^2^.

### 3.2. Genetic Polymorphism Analysis

The genotyping of CYP2C9 and OATP1B1 was performed for all 112 participants in the clinical trials. As a result of the genotyping of the CYP2C9 gene, 97, 12, and 3 subjects had *1*1, *1*3, and *1*13, respectively (frequencies: 86.61%, 10.71%, and 2.68%, respectively). These frequencies of CYP2C9 genotypes in this study were similar to those of CYP2C9 genotypes performed for a large number of Koreans reported in the past [30,33]. The frequencies of CYP2C9*1*1, *1*3, and *1*13 in previous reports [30,33] were 88.7–90.5%, 8.3–10.6%, and 0.4–0.9%, respectively. According to the genotypes of the *CYP2C9* gene, *1*1 was an extensive metabolizer (EM) and *1*3 and *1*13 were intermediate metabolizers (IMs). EM and IM meant normal and intermediate in the degree of metabolism by CYP2C9, respectively [16]. As a result of the genotyping of the SLCO1B1 gene, *1a*1a, *1a*1b, *1b*1b, *1a*15, *1b*15, and *15*15 were found in 11, 38, 37, 4, 19, and 3 subjects, respectively (frequencies: 9.82%, 33.93%, 33.04%, 3.57%, 16.96%, and 2.68%, respectively).

Depending on the genotypes of the SLCO1B1 gene, *1a*1a, *1a*1b, and *1b*1b were phenotypically classified as extensive transporters (ETs), *1a*15 and *1b*15 as intermediate transporters (ITs), and *15*15 as a poor transporter (PT) [34,35]. Here, ET, IT, and PT mean normal, intermediate, and low in the degree of transporter function, respectively [34]. The OATP1B1 genotyping results in this study were similar to previously reported SLCO1B1 haplotype frequencies. They were not significantly different from global OATP1B1 phenotype frequencies [34]. The fact that the *5 haplotype of the SLCO1B1 gene was rarely found in East Asian populations [34] was also consistent with the SLCO1B1 genotype frequency results in this study.

A total of six phenotypes could be classified by the combination of CYP2C9 and OATP1B1 phenotypes. Frequencies of ET/EM, ET/IM, IT/EM, IT/IM, PT/EM, and PT/IM were 71.43%, 5.36%, 15.18%, 5.36%, 0%, and 2.68%, respectively. Table 1 presents the genotyping results of CYP2C9 and OATP1B1 performed for 112 participants in this study.

### 3.3. Determination of Torsemide Serum Concentrations

To determine the concentrations of torsemide in serum samples, the method introduced in previous reports [20,21] was applied in this study. A total of 1344 serum samples were analyzed. The calibration curves of torsemide obtained from the serum samples showed an excellent linearity in the range of 0.02 to 10 μg/mL. The average calibration curve equation was as follows: Y (peak area ratio of torsemide and IS) = X (concentration of torsemide, μg/mL) × 0.493 + 0.0026. The R^2^ values of the calibration curves were very high, over 0.999. No significant interferences from the system or endogenous material peaks were identified. The peak spectrum of torsemide at the same retention time as the standard was also confirmed in the samples with a UV detector. This meant that torsemide could be selectively isolated and quantified from the human serum samples. Representative peak chromatograms of torsemide and IS quantified from the human serum samples are presented in Figure S2. The retention time of torsemide and IS were approximately 8.5 min and 10 min, respectively. Intra- and inter-batch accuracies for torsemide were 94.53–105.26% and 93.91–105.17%, respectively. Intra- and inter-batch analytical precisions for torsemide were within 10.43% and 9.50%, respectively (as coefficient of variation values). Here, both intra- and inter-batch accuracy and precision values were calculated using five replicates (*n* = 5). No significant carryover phenomenon was identified. Significant carryover meant that large residual analytes from the previous assay might appear in the next assay and affect quantitation. Recoveries of torsemide and IS from the human serum samples were both high (>90%) and consistent. The stabilities of torsemide and IS in human serum samples were both within ±15%. In incurred sample reanalysis, differences from the previous analysis were all within ±10%. As a result, the HPLC-UV method used in this study was sufficiently revalidated in terms of selectivity and specificity, linearity, precision, accuracy, carryover, recovery, stability, and incurred sample reanalysis of the assay. These verification results were in accordance with the standards proposed in the FDA’s Bioanalytical Method Validation guideline [32].

### 3.4. PK Results by NCA

Blood sampling time points for all subjects were established based on previously reported PK results for torsemide [20,21,22,23,24,25,26,27,28,29]. According to past reports [22,23,24,25,26,27,28,29], torsemide T_1/2_ in humans was approximately 1.7–3.8 h. Therefore, the blood sampling time after torsemide administration was set to 12 h, which was 3–4 times T_1/2_. The number of samplings was set to at least two points before reaching C_max_. Considering that the reported T_max_ of torsemide was approximately 1–2 h [20,21,22,23,24,25,26,27,28,29], blood sampling was performed at 0.5 h intervals until 2 h after the oral administration of torsemide. Table S1 summarizes the PK results of previous studies on torsemide used as reference for this study.

**Figure 1:**
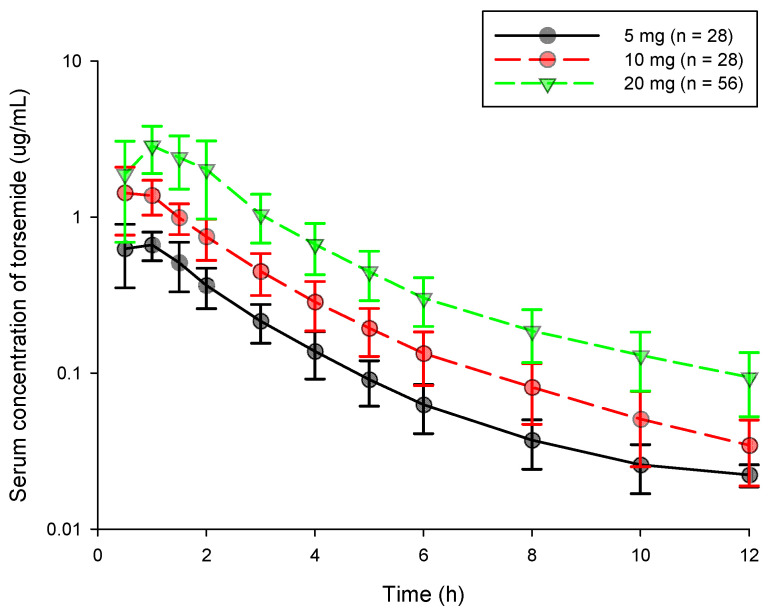
Serum concentration-time graph by dose according to oral torsemide administration in healthy Korean men. Black, red, and yellow-green points represent observations in 5, 10, and 20 mg torsemide administration groups, respectively. Vertical bar represents standard deviation (SD) at each point.

The serum concentration (log scaled)-time profiles obtained after oral administration of torsemide at 5, 10, and 20 mg for a total of 112 subjects are shown in Figure 1. It was confirmed that torsemide reached C_max_ within 1.5 h in blood after oral administration. After reaching C_max_ in the blood, it was confirmed that torsemide concentration gradually decreased in the form of a double slope. That is, the torsemide concentration in the blood rapidly decreased until about 1.5–6 h. It was then gradually eliminated after that. The serum concentration-time profiles according to doses of torsemide at 5, 10, and 20 mg showed a similar pattern without large differences among the dose groups.

**Figure 2:**
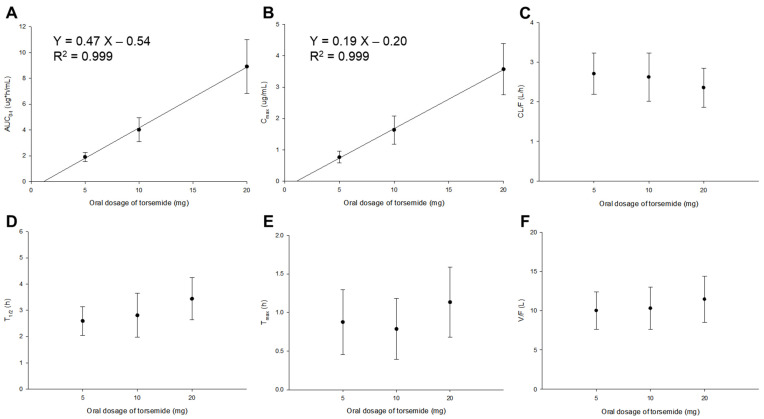
Graphs of changes in PK parameters (AUC_0–t_ (**A**); C_max_ (**B**); CL/F (**C**); T_1/2_ (**D**); T_max_ (**E**); V/F (**F**)) by dose according to oral administration of torsemide in healthy Korean men. PK parameters were calculated by NCA. Vertical bar represents the SD at each point.

Figure 2 presents the PK parameter results of torsemide by dose obtained with NCA for 112 subjects. The mean T_1/2_ and T_max_ values of torsemide were 2.59–3.44 h and 0.79–1.13 h, respectively. These were similar to previously reported PK results (T_1/2_ and T_max_ of 1.7–3.8 h and 0.5–2 h, respectively) following the oral administration of torsemide in healthy adult males (Table S1). The mean CL/F and V/F values of torsemide were 2.35–2.71 L/h and 10.00–11.44 L, respectively. These relatively large CL/F and V/F values suggested the extensive clearance and body distribution of torsemide from the bloodstream. According to a previous report [1], AUC and C_max_ increased linearly, with a high correlation from 2.5 to 200 mg dose after oral administration of torsemide in humans. This indicated the PK linearity of torsemide. Similarly, in this study, the PK linearity was confirmed according to the oral administration of torsemide at 5, 10, and 20 mg. There were no significant differences (*p* > 0.05 by one-way ANOVA) in the PK parameter values of T_1/2_, T_max_, CL/F, or V/F for each dose group (5, 10, 20 mg). It was confirmed that AUC_0–t_ and C_max_ increased linearly with increasing dose of 5, 10, and 20 mg. Taking the results of Figure 2 into consideration, the PK linearity was confirmed for torsemide at doses of 5 to 20 mg. These torsemide PK linearity results were the reason why nonlinear kinetic models were not considered when setting up the torsemide Pop-PK basic model in this study. Additionally, when the NCA results for each torsemide dose according to the phenotypes of CYP2C9 and OATP1B1 were checked, PK linearities were also confirmed, as shown in Figure 2. That is, in each group of EM and IM, there were no significant differences in T_1/2_, T_max_, CL/F, or V/F between the dose groups. In addition, AUC_0–t_ and C_max_ were dose-proportional. A similar trend was observed in the comparison of the PK parameter values in ET, IT, and PT. Figures S3 and S4 show the NCA results for each dose according to the CYP2C9 and OATP1B1 phenotype classifications, respectively.

Average values of AUC_0–t_/AUC_0–∞_ ratios in the 5, 10, and 20 mg torsemide dose groups were 94.58–96.35%. The mean C_max_ value in the torsemide 5 mg group was relatively high at 0.77 μg/mL. These high AUC_0–t_/AUC_0–∞_ and C_max_ values imply that a lower limit of quantification (LLOQ) of 0.02 μg/mL is a sufficient sensitivity for PK studies with serum samples obtained after the oral administration of torsemide to humans. They also implied that the setting of sampling points was appropriate in this torsemide PK study. The PK parameter values calculated using NCA (Figure 2) were referenced and used as initial values in the construction of the torsemide Pop-PK basic model.

The PK parameter values calculated for each phenotype of CYP2C9 and OATP1B1 by NCA and their comparison are presented in Figures S5 and S6, respectively. Dose-normalized AUC_0–t_ and C_max_ values and T_1/2_ were significantly (*p* < 0.05) lower in EM than in IM. However, CL/F was significantly (*p* < 0.05) lower in IM than in EM. On the other hand, V/F and T_max_ were not significantly different (*p* > 0.05) between EM and IM. Overall, the results shown in Figure S5 suggest that there would be quite a large difference in the in vivo metabolism of torsemide by CYP2C9 between the EM and IM groups. The PK parameters of dose-normalized AUC_0–t_ and C_max_ values and T_1/2_ among the ET, IT, and PT groups were significantly (*p* < 0.05) higher in PT. However, CL/F was lower in PT. On the other hand, V/F and T_max_ were not significantly different (*p* > 0.05) between the ET, IT, and PT groups. The results in Figure S6 were similar to those in Figure S5, showing that there were significant differences in PK parameters (CL/F, T_1/2_, dose-normalized AUC_0–t_ and C_max_) between the genotypes. The PK parameter values calculated by NCA for each phenotypic combination of CYP2C9 and OATP1B1 and their comparison are presented in Figure S7. The PK parameters of CL/F, T_1/2_, dose-normalized AUC_0–t_, and C_max_ values were significantly (*p* < 0.05) different between groups. This suggests that genetic polymorphisms in CYP2C9 and OATP1B1 might be validly involved in the in vivo metabolism and disposition of torsemide. However, the overall effect of the genetic polymorphism of CYP2C9 on the PK parameter (particularly related to metabolism) values was greater than that of OATP1B1. This was because the average values of CL/F, T_1/2_, dose-normalized AUC_0–t_, and C_max_ were significantly different according to EM and IM rather than ET, IT, or PT.

### 3.5. Pop-PK Model Analysis

As a basic compartment structure, the serum torsemide concentration data obtained after the oral administration of 5, 10, and 20 mg of torsemide to humans were well described with a two-compartment model. Although the number of parameters to be estimated increased by four when applied to the two-compartment model than in the one-compartment model, −2LL and AIC, the objective values indicating model fit, were significantly reduced. The decrease was much larger than 9.49 (based on *Chi-square distribution*; α = 0.05, df = 4). In the three-compartment application, −2LL and AIC were also increased, along with an increase in the total number of parameters. Therefore, it was appropriate for the two-compartment configuration to be the basic structure of the torsemide Pop-PK model. This was consistent with the fact that the plasma concentration data of torsemide were better explained by the two-compartment model than by the one-compartment model in a previously reported study [8].

It was better in terms of model fit if the lag-time was reflected in the in vivo absorption process following the oral administration of torsemide. Although the number of parameters to be estimated in the model increased by two more according to lag-time reflection, improved GOF was confirmed with significantly lower −2LL and AIC values (based on *Chi-square distribution*; α = 0.05, df = 2) than when lag-time was not reflected. Rates related to the absorption, distribution, and elimination of torsemide from each compartment were well described as first-order rates. The reason was that when the zero-order rate was applied, the −2LL and AIC values were about 500–1000 higher than when the first-order rate was applied. As a result, the PK parameters constituting the basic structural model of torsemide were as follows: oral absorption rate constant (K_a_), oral absorption lag-time (T_lag_), volume of distribution for the central compartment (V/F) and peripheral compartment (V_2_/F), and clearance for the central compartment (CL/F) and peripheral compartment (CL_2_/F; clearance between the central and peripheral compartments).

The residual error reflecting within-subject error was well explained by the proportional error model. Values of −2LL and AIC in the proportional error model were lower than those in the additive, log additive, mixed, and power models that were tried (without any change in the total number of parameters). This implied that the proportional error model was more suitable than the additive, log additive, mixed, and power models in explaining the within-subject variability of torsemide.

IIV was considered or excluded sequentially for parameters of T_lag_, K_a_, V/F, V_2_/F, CL/F, and CL_2_/F constituting the basic model of torsemide Pop-PK to determine whether exponential error IIV model application was appropriate (to each parameter). As a result, it was confirmed that −2LL and AIC values were significantly increased (based on *Chi-square distribution*; α = 0.01, df = 1) when IIV was not considered for the parameters of T_lag_, V/F, V_2_/F, CL/F, and CL_2_/F. On the other hand, when IIV was not considered for K_a_, it was confirmed that the −2LL and AIC values were significantly decreased along with the decrease in the total number of parameters (based on *Chi-square distribution*; α = 0.01, df = 1). This suggested that it was appropriate to only consider IIV for T_lag_, V/F, V_2_/F, CL/F, and CL_2_/F among T_lag_, K_a_, V/F, V_2_/F, CL/F, and CL_2_/F parameters. Therefore, in torsemide’s Pop-PK model, K_a_ was considered a typical value (tv) without reflecting IIV. A summary of the steps taken to develop the basic structural model of torsemide is presented in Table 2.

The PK parameter values obtained by NCA were analyzed for correlation with the collected potential covariates to search for covariates that could explain IIV in torsemide’s PK parameters. Potential covariates here meant the physicochemical analyses of 56 subjects orally administered with 20 mg torsemide and genetic information for CYP2C9 and OATP1B1 for all 112 subjects. The results of the correlation analysis between the physicochemical information treated as continuous data and the PK parameters of torsemide are presented in Figure S8. Those with relatively high correlation values among the results plotted for several candidate covariates and individual post hoc parameters are also shown in Figure S8. Positive correlations were found for CL/F-CrCl, V/F-total protein, V/F-BMI, CL/F-BMI, and CL/F-body weight. Negative correlations were found for CL/F-total protein, V/F-BSA, C_max_-BMI, and T_max_-body weight. However, their degrees of correlation were not high, with R^2^ values less than 0.09 (meaning approximately 30% correlation). Such a low degree of correlation implied that the correlation between the PK parameters of torsemide and the physicochemical covariates was not large. Therefore, it was difficult to find a valid covariate that could explain the PK variability of torsemide within the population. The results shown in Figure S8 were tried as covariates of relevant PK parameters in order to check whether the physicochemical parameters could be considered as covariates modelologically. The physicochemical parameter values were normalized to the median of observed value. These values were reflected in the model. As a result, it was confirmed that the physicochemical parameters collected in this study were not suitable as covariates that could effectively explain the PK variability of torsemide. This might be related to the fact that 52 subjects who received torsemide 20 mg were all normal adult males with a limited age range of 19 to 29 years. That is, effective correlations with PK parameters could not be confirmed because the fluctuation ranges of the values of various physiological and biochemical parameters such as the height, weight, BMI, and CrCl of individuals were very narrow.

Phenotypes classified based on the genotyping results of CYP2C9 and OATP1B1 were then attempted as covariates to explain the PK diversity of torsemide between individuals. Genetic phenotypes were treated as categorical data. The validity of the genetic information as covariates in the torsemide Pop-PK model was evaluated by the sequential application of PK parameters (excluding K_a_) in the basic structural model. The fit of the model was judged by the degree of change (with the number of parameters taken into account) of the OFV values relative to the torsemide’s base model (without covariates). Table 3 summarizes the stepwise search procedures based on OFV in the selection of the covariates for the final torsemide Pop-PK model. When the genetic polymorphism factors were applied to the basic model, not only was each phenotype applied to each parameter, but also the CYP2C9 and OATP1B1 phenotypes were applied to each parameter at the same time. When the genetic phenotypes of OATP1B1 and CYP2C9 were included in the basic model of torsemide as covariates for V_2_/F and T_lag_, there was a slight decrease in OFV. However, the decrease was less than the significance criterion of 3.84 (*p* < 0.05; one increase in the number of parameters) or 5.99 (*p* < 0.05; two increments in the number of parameters). The decrease in OFV (compared to the base model) was less than 3.84 when the CYP2C9 phenotype was reflected as a covariate of V/F, but was greater than 5.99 when OATP1B1 was considered as a covariate of V/F. When the OATP1B1 and CYP2C9 phenotypes were reflected in the CL/F and CL_2_/F covariates, the reduction in OFV (compared to the base model) was both greater than 3.84 or 5.99. Therefore, as a result of exploring the covariates by forward addition, it was modelologically appropriate to reflect the phenotypes of OATP1B1 as V/F covariates with OATP1B1 and CYP2C9 phenotypes as covariates of CL/F and CL_2_/F. This was considered a full model. For reconfirmation of the covariate validity, PK parameters and genetic phenotype relationships were removed step by step from the full model through a backward removal step. When CYP2C9 was not considered a covariate of CL/F or CL_2_/F, the OFV values were greater than 6.63 (*p* < 0.01; one increase in the number of parameters). When OATP1B1 was not considered a covariate of CL/F or CL_2_/F, the OFV values were increased to less than 9.21 (*p* < 0.01; two increments in the number of parameters). On the other hand, when the OATP1B1 phenotype was not reflected as a covariate of V/F, the increase in the OFV value was significant at 9.46. Therefore, in the Pop-PK model of torsemide, OATP1B1 as an effective covariate for V/F and CYP2C9 as an effective covariate for CL/F and CL_2_/F were finally considered.

**Figure 3:**
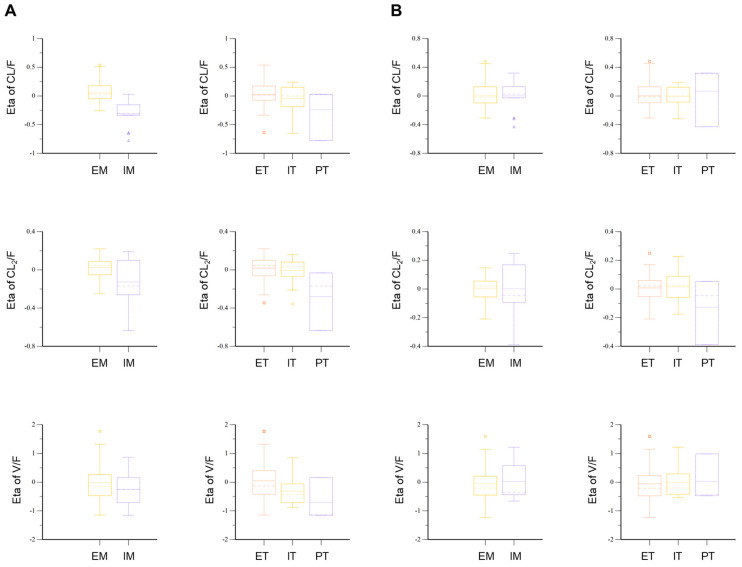
Eta plots of CL/F, CL_2_/F, and V/F parameters according to CYP2C9 and OATP1B1 in the base model (**A**) and the final model (**B**) for torsemide.

The final model had an OFV value of −2844.40, a decrease in the number of total parameters by four without a significant increase in OFV compared to the full model. Compared to the base model, the OFV of the final model was significantly reduced by about 78.54. This suggested that genetic polymorphisms in OATP1B1 and CYP2C9 were effective in explaining the interindividual PK variability of torsemide. The values of the PK parameters in humans estimated using the established torsemide Pop-PK model are presented in Table 4. The relative standard error (RSE) values of all parameters estimated in torsemide’s final Pop-PK model were 2.65–31.56%. The Eta shrinkage (%) values of T_lag_, V/F, V_2_/F, CL/F, and CL_2_/F were 2.79–38.53%. In the final model, the IIV values of V/F, CL/F, and CL_2_/F were 74.95%, 17.10%, and 14.90%, respectively. These IIV (%) values were approximately 5–10% lower than the IIV values in the torsemide base model. This indirectly suggested that it was effective to reflect the genetic polymorphisms of OATP1B1 and CYP2C9 as covariates of V/F, CL/F, and CL_2_/F in the final Pop-PK model of torsemide. This was because the decrease of IIV in the final model meant that the PK variability of torsemide was partially explained by the reflection of covariates in V/F, CL/F, and CL_2_/F. The covariate fit was also confirmed by comparing the Eta plot results of the torsemide base model and the final model. Before the genetic polymorphisms of CYP2C9 and OATP1B1 were reflected as covariates for CL/F, CL_2_/F, and V/F, the Eta values in the basic model were different between each phenotypic group (Figure 3). In particular, differences between groups were identified in the median and mean values of Eta for each phenotype. However, in the final model, the Eta values between each phenotype tended to be largely reduced compared to those in the base model (Figure 3). The median and mean values of the Eta of V/F for each phenotype of ET, IT, and PT were all close to zero. The median and mean values of Eta in CL/F and CL_2_/F for each phenotype in EM and IM were also close to zero. This suggested that by reflecting OATP1B1 as a covariate for V/F in the final model with CYP2C9 as a covariate for CL/F and CL_2_/F in the final model, the PK variability of torsemide between individuals could be explained more effectively than by the base model. Figure 3 shows the results of the Eta plots in the base model and the final model according to the OATP1B1 and CYP2C9 phenotypes.

The degree of association between V/F and the OATP1B1 phenotype was estimated to be −0.41 for IT and −0.65 for PT (Table 4). This implied that the V/F would be smaller in IT and PT than in ET. It was also smaller in PT than in IT. The degree of association between CL/F and the CYP2C9 phenotype was estimated to be 0.51. The degree of association between CL_2_/F and the CYP2C9 phenotype was estimated to be 0.37 (Table 4). This implied that CL/F and CL_2_/F would be larger in EM than in IM. The formulas of the finally established torsemide Pop-PK model were as follows:

V/F = tvV/F · (1 + dV/FdOATP1B1_IT_ · (OATP1B1_ET_ = 0 or OATP1B1_IT_ = 1)) or (1 + dV/FdOATP1B1_PT_ · (OATP1B1_ET_ = 0 or OATP1B1_PT_ = 1)) · exp(*ŋ*_V/F_) (1)

CL/F = tvCL/F · (1 + dCL/FdCYP2C9 · (CYP2C9_IM_ = 0 or CYP2C9_EM_ = 1)) · exp(*ŋ*_CL/F_) (2)

V_2_/F = tvV_2_/F · exp(*ŋ*_V2/F_) (3)

CL_2_/F = tvCL_2_/F · (1 + dCL_2_/FdCYP2C9 · (CYP2C9_IM_ = 0 or CYP2C9_EM_ = 1)) · exp(*ŋ*_CL2/F_) (4)

K_a_ = tvK_a_ (5)

T_lag_ = tvT_lag_ · exp(*ŋ*_Tlag_) (6)

In these formulas, dV/FdOATP1B1_IT_ and dV/FdOATP1B1_PT_ meant the degree of association between V/F and the OATP1B1 phenotypes IT and PT, respectively; dCL/FdCYP2C9 and dCL_2_/FdCYP2C9 meant the degrees of association between CL/F and CL_2_/F and the CYP2C9 phenotypes, respectively.

As a result of checking the correlations between the Eta of the parameters, significant relationships were not confirmed. In particular, the R^2^ values between the Eta values of V/F and V_2_/F and CL/F and CL_2_/F were less than 0.1, indicating no significant correlation with each other. Therefore, in this torsemide Pop-PK model, structurally, the Omega block (considering the correlation between the Eta parameters) was not established. Figure S9 shows the results of the correlation analysis between the Eta values of V/F, V_2_/F, CL/F, and CL_2_/F.

### 3.6. Pop-PK Model Interpretation

Consequently, the polymorphism of CYP2C9 in this torsemide Pop-PK model could effectively explain the interindividual diversity of CL/F and CL_2_/F. The model confirmed that the CL/F and CL_2_/F of torsemide were significantly faster in EM than in IM by approximately 51.0 and 36.5%, respectively. This suggested that when IM was confirmed through CYP2C9 genotyping, the treatment effect could be maintained while reducing the side effects of torsemide by lowering the dose of torsemide than in patients with EM. Moreover, the polymorphisms of OATP1B1 in this torsemide Pop-PK model could effectively explain the IIV of V/F. In other words, the model confirmed that the V/F of torsemide was significantly smaller in IT and PT by approximately 41.0% and 64.6%, respectively, than in ET. This could be explained by the fact that when the phenotype of OATP1B1 was ET, it could rapidly transport the substrate, torsemide, from the blood to the hepatocytes, so that the volume of distribution in the body was largely estimated. As the degree of torsemide transport from blood into hepatocytes decreased when IT and PT progressed, it could be explained that the volume of distribution of torsemide in the body was estimated to be low.

In addition, considering that the genetic polymorphisms of CYP2C9 and OATP1B1 could affect different parameters (CL/F and CL_2_/F to CYP2C9; V/F to OATP1B1) related to the PK diversity of torsemide, an interaction between the genetic factors could be estimated. As a result, it could be estimated that there was no interaction (related to torsemide’s PKs) between CYP2C9 and OATP1B1 in the body, unlike P-glycoprotein (P-gp) and CYP3A4/5 or CYP2D6 interplaying with the cell membrane permeation and metabolic processes [36,37]. These results were consistent with a previous report [38] showing that the genetic polymorphisms of OATP1B1 and CYP2C9 were presumed to influence the PKs of torsemide independently.

The significant effects of various physicochemical factors (such as height, weight, age, albumin, total proteins, BMI, BSA, ALP, AST, ALT, total bilirubin, cholesterol, BUN, GFR, creatinine), including CrCl, on torsemide PKs were not identified in this study. Considering that a previous report has shown that unmetabolized torsemide is excreted in the urine through the kidney [1], significant correlations between the CL/F of torsemide and CrCl suggesting the degree of renal function were explored. Considering that the reported protein binding of torsemide is over 99% [1,6], it was thought that the correlation between the total proteins (or albumin) and the V/F or CL/F of torsemide could be explored. However, the reason these physicochemical factors were not explored as effective covariates was because this torsemide PK study was conducted using healthy adults. That is, the physicochemical parameter values of the subjects did not differ largely from each other, as most of them fell within normal ranges. Therefore, it was difficult to find factors that could explain the difference in the PKs between individuals within normal physicochemical values. In addition, a previous report [1] has shown that the degree of renal function and the total plasma clearance of torsemide appear to be independent, consistent with the result of this Pop-PK analysis.

The ω^2^ estimate in the final model of the V/F associated with the body distribution of torsemide was 0.562 (with IIV of 74.95%), which was not small. In addition, the ω^2^ estimate in the final model of T_lag_ related to the oral absorption of torsemide was 0.341 (with IIV of 58.37%), which was not small, either. This suggests that IIV exists in the body distribution and oral absorption of torsemide and that there are still potential covariates that may explain the PK diversity of torsemide through further studies. Therefore, based on this study, it will be necessary to conduct research to explore the additional factors related to the variability of torsemide in its body distribution and oral absorption. The ε value representing the distribution of residuals was 0.136, and the RSE was 7.15%, which was not large.

This study was important in that it explored and presented the covariates that could effectively explain IIV in torsemide PKs. The final selected covariates are related to torsemide drug therapy in the clinical setting. Thus, this study has a very important meaning in that it can enable more effective treatment than existing ones. That is, taking into account the patient’s CYP2C9 and OATP1B1 genotypes, the dose and administration interval of torsemide used for diuresis may be appropriately increased or decreased. However, in order to clarify the specific dose setting and administration control of torsemide, further studies on the pharmacodynamic aspects of torsemide for patients are needed based on this Pop-PK modeling study.

### 3.7. Pop-PK Model Evaluation

**Figure 4:**
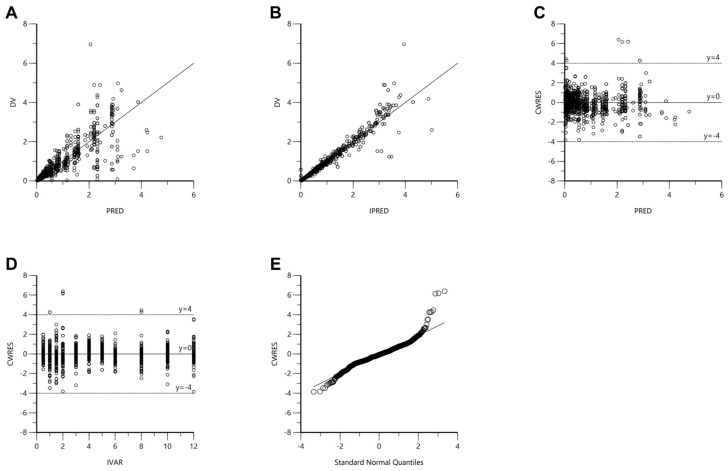
GOF plot results for the final torsemide Pop-PK model. DV versus PRED (**A**). DV versus IPRED (**B**). CWRES versus PRED (**C**). CWRES versus IVAR (**D**). CWRES versus quantile–quantile plot of components (**E**).

The established torsemide Pop-PK model was comprehensively evaluated through GOF plots, bootstrapping, VPC, NPDE, and external validation. The GOF plot results for the final Pop-PK model of torsemide are presented in Figure 4. The torsemide concentration values (in the population or individual) predicted by the Pop-PK model showed relatively good agreement with experimentally obtained observations. The CWRES values were well distributed symmetrically around zero, and the distribution was random with no notably large specific bias. At most points of PRED or IVAR, the CWRES values did not deviate significantly from ±4. The quantile–quantile plot of the CWRES component was close to a straight line, with symmetric *X*- and *Y*-axes. As a result, it was confirmed through the GOF plot results presented in Figure 4 that the finally established torsemide Pop-PK model did not have any serious graphical problems.

Table 5 shows the bootstrapping results for the finally established torsemide Pop-PK model. As a result, all parameter values estimated from the torsemide final model were within the 95% CIs of the bootstrap analysis results with 1000 iterations. The reason why the 95% CI interval of dV/FdOATP1B1_PT_ was quite wide, from −1.000 to −0.096, is thought to be because the number of observations in PT (as 2.68%) was relatively small compared to that of ET (as 76.79%) and IT (as 20.53%), and the torsemide concentration differences between the subjects were large in PT. Therefore, dV/FdOATP1B1_PT_ would have widened the 95% CI interval in the process of parameter estimation through iterative data set reconstruction. This suggested that the V/F variability in PT was relatively high. The parameter estimates of the final model were close to the bootstrap-estimated median, and the differences between them were within 20%. The reproducibility and robustness of the finally established torsemide Pop-PK model were confirmed by bootstrap analysis.

**Figure 5:**
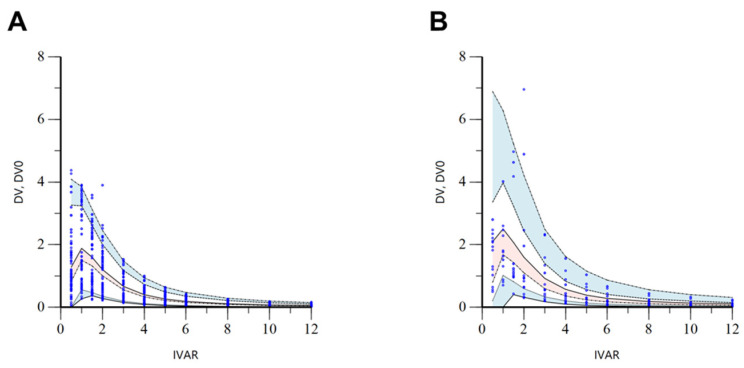
Stratified VPC results by EM (**A**) and IM (**B**) phenotypes of CYP2C9 for the final torsemide Pop-PK model. The dots on the graph represent observations. The black dashed lines mean the 95th, 50th, and 5th percentiles of predicted concentrations, respectively. Blue shaded areas mean 95% CIs for the predicted 95th and 5th percentiles. Red shaded area means 95% CI for the predicted 50th percentile. IVAR (*X*-axis) means the time after oral torsemide administration. DV and DV0 (*Y*-axis) are observed concentrations of torsemide in the serum and concentrations predicted by the model, respectively.

**Figure 6:**
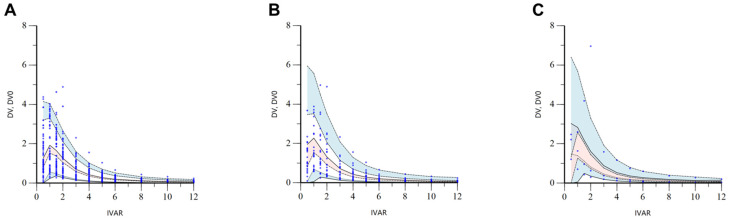
Stratified VPC results by ET (**A**), IT (**B**), and PT (**C**) phenotypes of OATP1B1 for the final torsemide Pop-PK model. The dots on the graph represent observations. The black dashed lines mean the 95th, 50th, and 5th percentiles of predicted concentrations, respectively. Blue shaded areas mean 95% CIs for the predicted 95th and 5th percentiles. Red shaded area means 95% CI for the predicted 50th percentile. IVAR (*X*-axis) means the time after oral torsemide administration. DV and DV0 (*Y*-axis) are observed concentrations of torsemide in the serum and concentrations predicted by the model, respectively.

The VPC results for the finally established torsemide Pop-PK model are presented in Figure S10. Most of the observation values (more than 90%) of torsemide were well distributed within 90% prediction intervals (of 5–95%) of prediction values. These VPC results suggested that torsemide’s Pop-PK model described the overall experimental data relatively well. Nevertheless, the reason that some observations were outside the VPC region in the high concentration interval was probably due to the considerable PK variability of torsemide between individuals. PK diversity was implicated by genetic polymorphisms in CYP2C9 and OATP1B1. The VPC results stratified according to the CYP2C9 phenotype are presented in Figure 5. It was confirmed through VPC that the concentration of torsemide in the serum was more rapidly decreased in the EM group than in the IM group. C_max_ values were also lower in EM than in IM. The VPC results stratified according to the OATP1B1 phenotype are presented in Figure 6. It was confirmed through VPC that the concentration of torsemide in the serum was low and rapidly decreased in the ET group, and that the concentration of torsemide in the serum was high and slowly decreased in the IT and PT groups. Regarding CYP2C9 and OATP1B1 being reflected as effective covariates in the parameters of CL/F, CL_2_/F, and V/F, the results of Figure 5 and Figure 6 well explained the associations between them.

**Figure 7:**
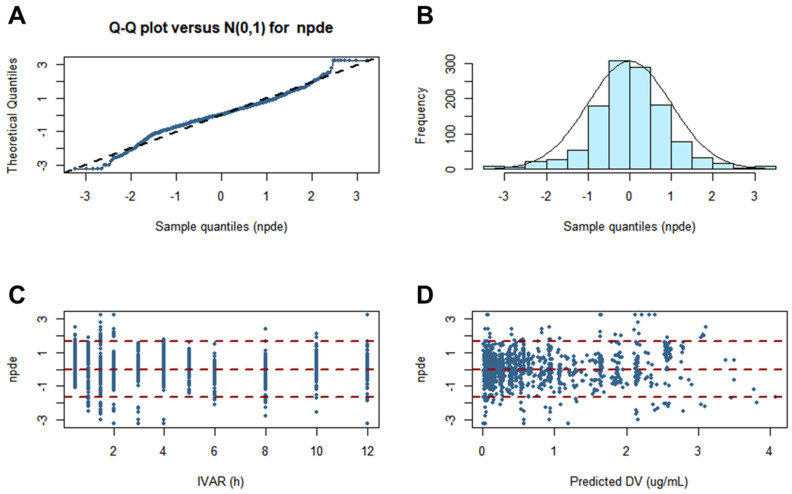
Graphs of NPDE results for the final torsemide Pop-PK model. Theoretical N (0, 1) distribution versus quantile–quantile plots of NPDE (**A**). Histogram showing the density of the standard Gaussian distribution and the distribution of superimposed NPDE (**B**). Scatter plot of the NPDE versus IVAR (**C**). Scatterplot of the NPDE versus predicted DV (**D**).

Performing a normality test of the model via NPDE was necessary. This is because NPDE is an analysis that considers the overall predictive distribution of each individual observation and handles multiple observations within one subject. The assumption of a normal distribution for the difference between observations and predictions was sufficiently acceptable. Furthermore, the normality of the NPDE was confirmed in histogram and quantile–quantile plots. The NPDE analysis results for the finally established torsemide Pop-PK model are presented in Figure 7.

**Figure 8:**
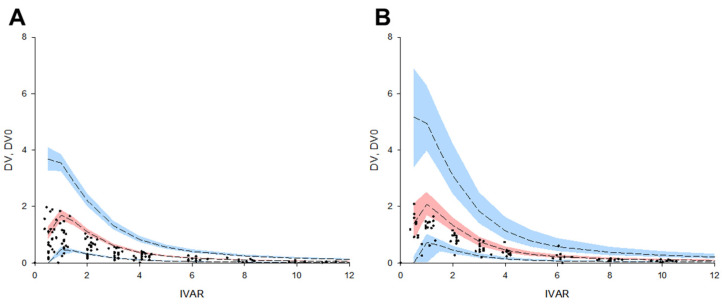
External validation results using the VPC of torsemide’s final Pop-PK model and external data set [8]. Data were stratified with EM (**A**) and IM (**B**) CYP2C9 phenotypes. The dots on the graph represent observations. The black dashed lines mean the 95th, 50th, and 5th percentiles of predicted concentrations, respectively. Blue shaded areas mean 95% CIs for the predicted 95th and 5th percentiles. Red shaded area means 95% CI for the predicted 50th percentile. IVAR (*X*-axis) means the time after oral torsemide administration. DV and DV0 (*Y*-axis) are observed concentrations of torsemide in the serum and concentrations predicted by the model, respectively.

Most observations of subjects included in the external validation data set were superimposed on the final torsemide Pop-PK model VPC plots. That is, the external data obtained after the oral administration of 10 mg of torsemide [8] were superimposed in the VPC results modeled by integrating the data of the torsemide 5, 10, and 20 mg dose groups. More than 90% of all data for EM and IM were included in the VPC plots for EM and IM, respectively. The observed and predicted medians were close to each other. This suggested that the finally established torsemide Pop-PK model predicted the PK characteristics according to the CYP2C9 phenotype (as EM and IM) well. The reason the results [8] were set as the external validation data set was because their study was suitable for confirming the degree of PK prediction according to the CYP2C9 genetic polymorphism of the torsemide Pop-PK model finally established in this study. In the previous report [8], the torsemide plasma concentration profiles were stratified according to the genotypes of CYP2C9 (group 1: CYP2C9*1*1, *1*2, and *2*2; group 2: CYP2C9*1*3 and *2*3; group 3: CYP2C9*3*3). The results stratified into groups 1 and 2 in the past report [8] could be considered as external validation data sets for EM and IM, respectively. This is because the result of a previous report [8] was that the torsemide PKs differed according to the number of alleles in CYP2C9*3 (as *3 increases from 0 to 1, 2, the plasma torsemide concentration increases), and the differences in the torsemide PKs associated with CYP2C9*2 were not significant [8]. Therefore, the previous report’s classification (as groups 1 and 2) [8] was predicted and applied to roughly correspond to EM and IM in this study. Figure 8 shows the results of the external validation performed and stratified according to the CYP2C9. Although racial differences exist and the CYP2C9 genotypes are not completely identical (between the previous study [8] and this study), the previous data [8] were sufficiently applicable as part of the validation based on approximate CYP2C9 phenotypic classification. As a result of the comprehensive evaluation of the finally established torsemide Pop-PK model, all verification results were acceptable without any major problems.

### 3.8. Reconfirmation of Torsemide Pop-PK Model

Phoenix NLME and NONMEM are representative software used for Pop-PK modeling worldwide. Therefore, NONMEM was applied for the reconfirmation of the torsemide Pop-PK model in this study. For torsemide Pop-PK modeling, the results of Phoenix NLME and NONMEM were almost similar without showing any significant difference. The model structure and equations were the same as in Phoenix NLME. The difference between the values from Phoenix NLME and NONMEM in estimates of parameters were almost 0%. For the RSE (%) and IIV (%) of the parameters, the differences between the values from Phoenix NLME and NONMEM were all less than ±0.1. This reconfirmed that the Pop-PK model of torsemide established in this study had robustness and reproducibility. In addition, it implied that the modeling software had little effect on the estimation of the PK parameter values within the population. Table 6 presents the results for the parameters calculated using NONMEM and their differences compared to the values estimated by Phoenix NLME.

## 4. Conclusions

In this study, a novel human Pop-PK model for torsemide was developed and validated. To the best of our knowledge, the studies presenting key contents and verification results focusing on torsemide Pop-PK modeling have not been reported yet. This study is the first to report a torsemide Pop-PK model in Koreans. The serum concentration-time profiles for torsemide in healthy Korean men were well described by a two-compartment model with delayed oral absorption (presented as lag-time). The OATP1B1 and CYP2C9 genetic polymorphisms were identified as effective covariates for the V/F, CL/F, and CL_2_/F of torsemide. Associations between genetic polymorphisms and PK parameters were reflected in the final Pop-PK model of torsemide. The developed model was fully validated. Consequently, the Pop-PK model study confirmed that OATP1B1 and CYP2C9 genetic polymorphisms could explain a significant degree of IIV in the PKs of torsemide. In addition, from the covariate effects of the CYP2C9 and OATP1B1 genetic polymorphisms on the different PK parameters of torsemide, it could be inferred that torsemide’s cell membrane permeation process by OATP1B1 and the metabolic process by CYP2C9 can independently affect systemic PKs without mutual in vivo interplay. There was no significant difference in the parameter estimates between Phoenix NLME and NONMEM modeling software. The results of this study are expected to be used as an important reference for improving the model and for exploring other effective covariates related to torsemide’s PK diversity within the population in the future. In addition, it is expected that the therapeutic effect of torsemide can be improved while reducing clinical side effects by adjusting the dosage and use of torsemide in consideration of the different genotypes of individual patients.

## Figures and Tables

**Table 1:** Genotype ratio and phenotypic classification of CYP2C9 and OATP1B1 in healthy Korean men (*n* = 112) who received torsemide orally.

CYP2C9							
Genotype		*1*1	*1*3	*1*13			
Subject No		97 (86.61%)	12 (10.71%)	3 (2.68%)			
Phenotype		EM	IM	IM			
**OATP1B1**							
Genotype		*1a*1a	*1a*1b	*1b*1b	*1a*15	*1b*15	*15*15
Subject No		11 (9.82%)	38 (33.93%)	37 (33.04%)	4 (3.57%)	19 (16.96%)	3 (2.68%)
Phenotype		ET	ET	ET	IT	IT	PT
**OATP1B1/CYP2C9**							
Genotype	OATP1B1	*1a*1a, *1a*1b, *1b*1b	*1a*1a, *1a*1b, *1b*1b	*1a*15, *1b*15	*1a*15, *1b*15	*15*15	*15*15
	CYP2C9	*1*1	*1*3, *1*13	*1*1	*1*3, *1*13	*1*1	*1*3, *1*13
Subject No		80 (71.43%)	6 (5.36%)	17 (15.18%)	6 (5.36%)	0 (0%)	3 (2.68%)
Phenotype		ET/EM	ET/IM	IT/EM	IT/IM	PT/EM	PT/IM

**Table 2:** Steps used for building a basic structural model of torsemide.

Model	Description	nParameter	−2LL	AIC	△−2LL	△AIC	Compared with
**Compartment model**							
01	1-compartment	7	−1106.512	−1092.512	-	-	-
02 *	2-compartment	11	−2201.649	−2179.649	−1095.14	−1087.14	01
**Absorption model**							
02	No T_lag_	11	−2201.649	−2179.649	-	-	-
03 *	With T_lag_	13	−2757.066	−2731.066	−555.42	−551.42	02
**Residual error model**							
03 *	Proportional	13	−2757.066	−2731.066	-	-	-
04	Additive	13	363.99	389.99	3121.06	3121.06	03
05	Log additive	13	−11.59	14.41	2745.48	2745.48	03
06	Mixed	14	−2529.50	−2501.50	227.57	229.57	03
07	Power	13	528.70	554.70	3285.77	3285.77	03
**IIV model**							
08 *	Remove IIV on K_a_	12	−2765.86	−2741.86	−8.80	−10.80	03
09	Remove IIV on V/F	12	−2530.69	−2506.69	226.37	224.37	03
10	Remove IIV on V_2_/F	12	−2705.07	−2643.98	52.00	87.09	03
11	Remove IIV on CL/F	12	−1834.89	−1810.89	922.18	920.18	03
12	Remove IIV on CL_2_/F	12	−2729.3692	−2705.3692	27.70	25.70	03
13	Remove IIV on T_lag_	12	−2477.5333	−2453.5333	279.53	277.53	03

*, the selected model in each step. nParameter, the total number of parameters applied to the model.

**Table 3:** Stepwise selection of covariates for the Pop-PK model of torsemide.

Model	Description	Summary	OFV	△OFV	Compared with	nParameter
1	Base model	-	−2765.86	-	-	12
**Forward addition**						
2	OATP1B1 on V/F	-	−2775.354	−9.490	Base model	14
3	OATP1B1 on V_2_/F	-	−2766.150	−0.286	Base model	14
4	OATP1B1 on CL/F	-	−2772.458	−6.594	Base model	14
5	OATP1B1 on CL_2_/F	-	−2778.904	−13.040	Base model	14
6	OATP1B1 on T_lag_	-	−2765.983	−0.119	Base model	14
7	CYP2C9 on V/F	-	−2767.611	−1.747	Base model	13
8	CYP2C9 on V_2_/F	-	−2768.067	−2.203	Base model	13
9	CYP2C9 on CL/F	-	−2813.517	−47.652	Base model	13
10	CYP2C9 on CL_2_/F	-	−2781.570	−15.706	Base model	13
11	CYP2C9 on T_lag_	-	−2765.885	−0.021	Base model	13
12	OATP1B1 on V/F, CL/F	-	−2782.070	−6.716	Model 2	16
13	OATP1B1 on V/F, CL/F, CL_2_/F	-	−2797.111	−15.041	Model 12	18
14	OATP1B1 on V/F, CL/F, CL_2_/F CYP2C9 on CL/F	-	−2838.7158	−41.605	Model 13	19
15	OATP1B1 on V/F, CL/F, CL_2_/F CYP2C9 on CL/F, CL_2_/F	Full model	−2850.9748	−12.259	Model 14	20
**Backward removal**						
16	OATP1B1 on V/F, CL/F, CL_2_/F CYP2C9 on CL_2_/F	Full model deleted CYP2C9 on CL/F	−2779.106	71.8688	Model 15	19
17	OATP1B1 on V/F, CL/F, CL_2_/F CYP2C9 on CL/F	Full model deleted CYP2C9 on CL_2_/F	−2837.9679	13.0069	Model 15	19
18	OATP1B1 on V/F, CL/F CYP2C9 on CL/F, CL_2_/F	Full model deleted OATP1B1 on CL_2_/F	−2844.7793	6.1955	Model 15	18
19	OATP1B1 on V/F, CL_2_/F CYP2C9 on CL/F, CL_2_/F	Full model deleted OATP1B1 on CL/F	−2850.8641	0.1107	Model 15	18
20	OATP1B1 on CL/F, CL_2_/F CYP2C9 on CL/F, CL_2_/F	Full model deleted OATP1B1 on V/F	−2841.5173	9.4575	Model 15	18
21 *	OATP1B1 on V/F CYP2C9 on CL/F, CL_2_/F	Full model deleted OATP1B1 on CL/F, CL_2_/F	−2844.404	6.5708	Model 15	16

*, the finally selected model. nParameter, the total number of parameters applied to the model.

**Table 4:** Parameter values of the finally established human Pop-PK model for torsemide.

Parameter	Estimate	SE	RSE (%)	Shrinkage (%)	IIV (%)
**Final model**					
tvV/F (L)	1.027	0.081	7.92	-	-
tvCL/F (L/h)	1.740	0.073	4.19	-	-
tvV_2_/F (L)	3.914	0.104	2.65	-	-
tvCL_2_/F (L/h)	0.828	0.056	6.71	-	-
tvK_a_ (1/h)	0.861	0.025	2.93	-	-
tvT_lag_ (h)	0.274	0.018	6.64	-	-
dV/FdOATP1B1_IT_	−0.410	0.129	31.54	-	-
dV/FdOATP1B1_PT_	−0.646	0.204	31.56	-	-
dCL/FdCYP2C9_EM_	0.510	0.063	12.37	-	-
dCL_2_/FdCYP2C9_EM_	0.365	0.056	15.23	-	-
ω^2^v_/F_	0.562	0.070	12.45	21.040	74.949
ω^2^_CL/F_	0.029	0.004	13.34	2.790	17.098
ω^2^_V2/F_	0.036	0.007	20.74	23.780	18.938
ω^2^_CL2/F_	0.022	0.006	25.28	38.530	14.899
ω^2^_Tlag_	0.341	0.079	23.16	23.370	58.367
ε	0.136	0.010	7.15	-	-

SE, standard error; RSE, relative standard error; IIV, inter-individual variability.

**Table 5:** Bootstrap results of the finally established human Pop-PK model for torsemide.

Parameter	Final Model		Bootstrap (*n* = 1000)	
	Estimate	95% CI	Median	95% CI
tvV/F (L)	1.027	0.868–1.187	1.042	0.861–1.233
tvCL/F (L/h)	1.740	1.597–1.884	1.733	1.473–2.005
tvV_2_/F (L)	3.914	3.710–4.118	3.892	3.698–4.131
tvCL_2_/F (L/h)	0.828	0.719–0.937	0.826	0.667–1.102
tvK_a_ (1/h)	0.861	0.811–0.910	0.859	0.818–0.919
tvT_lag_ (h)	0.274	0.238–0.309	0.278	0.226–0.324
dV/FdOATP1B1_IT_	−0.410	−0.663–−0.156	−0.409	−0.586–−0.203
dV/FdOATP1B1_PT_	−0.646	−1.046–−0.246	−0.664	−1.000–−0.096
dCL/FdCYP2C9_EM_	0.510	0.386–0.634	0.505	0.310–0.786
dCL_2_/FdCYP2C9_EM_	0.365	0.256–0.473	0.357	0.093–0.659
ω^2^v_/F_	0.562	0.425–0.699	0.536	0.357–0.715
ω^2^_CL/F_	0.029	0.022–0.037	0.028	0.021–0.035
ω^2^_V2/F_	0.036	0.021–0.050	0.035	0.016–0.053
ω^2^_CL2/F_	0.022	0.011–0.033	0.019	0.002–0.036
ω^2^_Tlag_	0.341	0.186–0.495	0.356	0.089–0.623
ε	0.136	0.117–0.156	0.137	0.117–0.150

CI, confidence interval.

**Table 6:** Parameter values of the finally established human Pop-PK model for torsemide using NONMEM software and comparison with values from Phoenix NLME.

Parameter	Estimate *	NONMEM−NLME Difference (%) of Estimate	RSE (%) *	NONMEM−NLME Difference of RSE	IIV (%) *	NONMEM−NLME Difference of IIV
**Final model**						
tvV/F (L)	1.027	0.00	8.04	0.12	-	-
tvCL/F (L/h)	1.740	0.00	4.32	0.13	-	-
tvV_2_/F (L)	3.914	0.00	2.68	0.03	-	-
tvCL_2_/F (L/h)	0.828	0.00	6.80	0.09	-	-
tvK_a_ (1/h)	0.861	0.00	2.94	0.01	-	-
tvT_lag_ (h)	0.274	0.00	6.66	0.02	-	-
dV/FdOATP1B1_IT_	−0.410	0.00	31.54	0.00	-	-
dV/FdOATP1B1_PT_	−0.646	0.00	31.59	0.03	-	-
dCL/FdCYP2C9_EM_	0.510	0.00	12.39	0.02	-	-
dCL_2_/FdCYP2C9_EM_	0.365	0.00	15.29	0.06	-	-
ω^2^_V/F_	0.562	0.00	12.47	0.02	74.967	0.02
ω^2^_CL/F_	0.029	0.00	13.38	0.04	17.029	−0.07
ω^2^_V2/F_	0.036	0.00	20.78	0.04	18.974	0.04
ω^2^_CL2/F_	0.022	0.00	25.91	0.63	14.832	−0.07
ω^2^_Tlag_	0.341	0.00	23.17	0.01	58.395	0.03
ε	0.136	0.00	7.15	0.00	-	-

*, values calculated using NONMEM. RSE, relative standard error; IIV, inter-individual variability.

## Data Availability

The data presented in this study are available in the article and Supplementary Materials.

## Supplementary Materials

The following supporting information can be downloaded at: https://www.mdpi.com/article/10.3390/pharmaceutics14040771/s1, Figure S1: Chemical structures of torsemide (A) and furosemide (B) used as IS; Figure S2: Representative peak chromatograms of torsemide and IS from human serum samples; Figure S3: Graphs of changes in PK parameters (AUC_0–t_ (A); C_max_ (B); CL/F (C); T_1/2_ (D); T_max_ (E); V/F (F)) by dose and CYP2C9 phenotype according to oral administration of torsemide in healthy Korean men. PK parameters were calculated by NCA. Vertical bar represents the SD at each point; Figure S4: Graphs of changes in PK parameters (AUC_0–t_ (A); C_max_ (B); CL/F (C); T_1/2_ (D); T_max_ (E); V/F (F)) by dose and OATP1B1 phenotype according to oral administration of torsemide in healthy Korean men. PK parameters were calculated by NCA. Vertical bar represents the SD at each point; Figure S5: Graphs of changes in PK parameters (dose normalized AUC_0–t_ (A); CL/F (B); V/F (C); T_1/2_ (D); dose normalized C_max_ (E); T_max_ (F)) by CYP2C9 phenotype according to oral administration of torsemide in healthy Korean men. PK parameters were calculated by NCA. Vertical bar represents the SD; Figure S6: Graphs of changes in PK parameters (dose normalized AUC_0–t_ (A); CL/F (B); V/F (C); T_1/2_ (D); dose normalized C_max_ (E); T_max_ (F)) by OATP1B1 phenotype according to oral administration of torsemide in healthy Korean men. PK parameters were calculated by NCA. Vertical bar represents the SD; Figure S7: Graphs of changes in PK parameters (dose normalized AUC_0–t_ (A); CL/F (B); V/F (C); T_1/2_ (D); dose normalized C_max_ (E); T_max_ (F)) by CYP2C9 and OATP1B1 phenotypes according to oral administration of torsemide in healthy Korean men. PK parameters were calculated by NCA. Vertical bar represents the SD; Figure S8: Correlation analysis graph between physiological or biochemical parameter values and PK parameter values (CrCl-CL/F (A); total protein-CL/F (B); total protein-V/F (C); BSA-V/F (D); BMI-V/F (E); BMI-CL/F (F); body weight-CL/F (G); BMI-C_max_ (H); body weight-T_max_ (I)) in healthy Korean males orally administered 20 mg torsemide (*n* = 56). PK parameters were calculated by NCA. CrCl was calculated based on the Cockcroft–Gault equation ((140-age) × body weight (kg)/serum creatinine (mg/dL) × 72). BMI was calculated based on the Kaup index (body weight (kg)/height^2^ (m^2^)). BSA was calculated based on the Mosteller formula $(\sqrt{\left(\mathrm{height}\;\left(\mathrm{cm}\right)\times \;\mathrm{weight}\;\left(\mathrm{kg}\right)/3600\right)}$); Figure S9: Eta plots between CL/F, CL_2_/F and V/F, V_2_/F in the final model for torsemide; Figure S10: VPC of the final model for torsemide without any stratification. Observed concentrations were depicted by dots. The 95th, 50th, and 5th percentiles of the predicted concentrations are represented by black dashed lines. The 95% confidence intervals (CIs) for the predicted 5th and 95th percentiles are represented by blue shaded regions. The 95% CIs for the predicted 50th percentiles are represented by red shaded regions. IVAR (*X*-axis) means the time after oral torsemide administration. DV and DV0 (*Y*-axis) are observed concentrations of torsemide in the serum and concentrations predicted by the model, respectively; Table S1: Summary of previously reported PK parameter values based on NCA following oral torsemide administration in healthy men; Methods for determining genotypes; Assay conditions to determine serum torsemide concentration. References [39,40,41] are cited in the supplementary materials.

## Abbreviations

PK	Pharmacokinetic
Pop-PK	Population pharmacokinetic
CrCl	Creatinine clearance
BSA	Body surface area
BMI	Body mass index
ALP	Alkaline phosphatase
AST	Aspartate transaminase
ALT	Alanine transaminase
GFR	Glomerular filtration rate
BUN	Blood urea nitrogen
HPLC	High-performance liquid chromatography
UV	Ultraviolet
HPLC-UV	High-performance liquid chromatography-ultraviolet
PCR-RFLP	Polymerase chain reaction–restriction fragment length polymorphism
SD	Standard deviation
ANOVA	Analysis of variance
tv	Typical value
NCA	Non-compartment analysis
AIC	Akaike’s information criterion
AUC	Area under the curve
IIV	Inter-individual variability
OFV	Objective function value
SE	Standard error
RSE	Relative standard error
VPC	Visual predictive check
NPDE	Normalized prediction distribution error
FOCE	First-order conditional estimates
NLME	Nonlinear mixed-effects
