# Supplementary material for: Population Pharmacokinetic (Pop-PK) Analysis of Torsemide in Healthy Korean Males Considering CYP2C9 and OATP1B1 Genetic Polymorphisms
Source: Pharmaceutics. 2022 Apr 1;14(4):771. doi: 10.3390/pharmaceutics14040771 (PMC9028991; doi:10.3390/pharmaceutics14040771)
Supplement: Supplementary file 1 [file pharmaceutics-14-00771-s001.zip › pharmaceutics-1655838-supplementary.pdf]

Supplementary Information

Population pharmacokinetic (Pop-PK) analysis of torsemide in healthy Korean males considering CYP2C9 and OATP1B1 genetic polymorphisms

Seung-Hyun Jeong, Ji-Hun Jang, Hea-Young Cho and Yong-Bok Lee

Table S1. Summary of previously reported PK parameter values based on NCA following oral torsemide administration in healthy men.

References	Subjects (healthy humans; male)	PK parameters					
		$T_{1/2}$ (h)	T_{max} (h)	C_{max} (μg/mL)	AUC_{0-t} (h·μg/mL)	CL/F (L/h)	V/F (L)
**Lesne et al., (1988) [1]**	n = 6, 20 mg dose	2.8 (0.3)[b]	0.5-1.5	0.8-3.2	5.7 (0.3)[b]	-[a]	13.7 (2.1)[b]
Cho et al., (2005) [2]	n = 28, 20 mg dose	-[a]	0.96 (0.36)[b]	3.52 (0.68)[b]	8.42 (1.60)[b]	-[a]	-[a]
Kang et al., (2013) [3]	n = 28, 10 mg dose	-[a]	0.79 (0.40)[b]	1.64 (0.45)[b]	3.87 (0.86)[b]	-[a]	-[a]
Vargo et al., (1995) [4]	n = 16, 10 mg dose	3.5 (1.2)[b]	0.86 (0.2)[b]	1.3 (0.1)[b]	3.7 (1.7)[b]	-[a]	-[a]
Lameire et al., (1988) [5]	n = 4, 20 mg dose	1.7 (0.6)[b]	1.5-2	2.8 (0.6)[b]	-[a]	-[a]	-[a]
Neugebauer et al., (1988) [6]	n = 9, 20 mg dose	3.7 (1.2)[b]	1.0 (0.5)[b]	2.79 (0.87)[b]	6.42 (1.59)[b]	-[a]	-[a]

Neugebauer et al., (1988) [6]	n = 9, 40 mg dose	3.2 (0.7)[b]	0.9 (0.3)[b]	5.02 (0.79)[b]	12.52 (2.09)[b]	-[a]	-[a]
Barr et al., (1990) [7]	n = 12, 5 mg dose	3.8 (0.7)[b]	1.0 (0.1)[b]	0.54 (0.11)[b]	1.77 (0.44)[b]	-[a]	-[a]
Barr et al., (1990) [7]	n = 11, 5 mg dose	2.4 (0.6)[b]	1.0 (0.4)[b]	0.56 (0.07)[b]	1.50 (0.41)[b]	-[a]	-[a]
Barr et al., (1990) [8]	n = 12, 2.5 mg dose	3.7 (2.6)[b]	1.1 (0.3)[b]	0.29 (0.03)[b]	0.91 (0.45)[b]	-[a]	-[a]
Barr et al., (1990) [8]	n = 12, 5 mg dose	3.5 (2.0)[b]	1.0 (0.3)[b]	0.65 (0.11)[b]	1.73 (0.57)[b]	-[a]	-[a]
Barr et al., (1990) [8]	n = 12, 10 mg dose	3.5 (1.2)[b]	0.9 (0.2)[b]	1.27 (0.13)[b]	3.67 (1.65)[b]	-[a]	-[a]
Kramer et al., (1995) [9]	n = 14, 10 mg dose	3.6 (1.0)[b]	0.9 (0.4)[b]	1.47 (0.20)[b]	3.36 (0.86)[b]	-[a]	-[a]
Kramer et al., (1993) [10]	n = 11, 10 mg dose	3.6 (1.3)[b]	1.3 (0.3)[b]	1.51 (0.32)[b]	3.87 (0.77)[b]	-[a]	-[a]

[a], information not provided in those reports.

[b], standard deviation (SD).

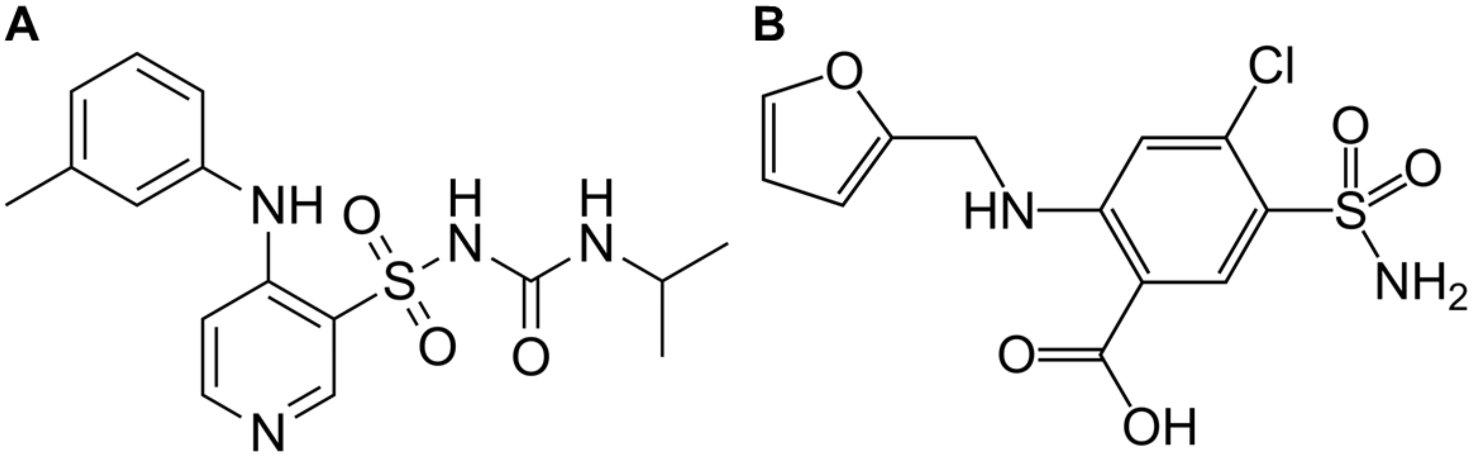

Figure S1. Chemical structures of torsemide (A) and furosemide (B) used as IS.

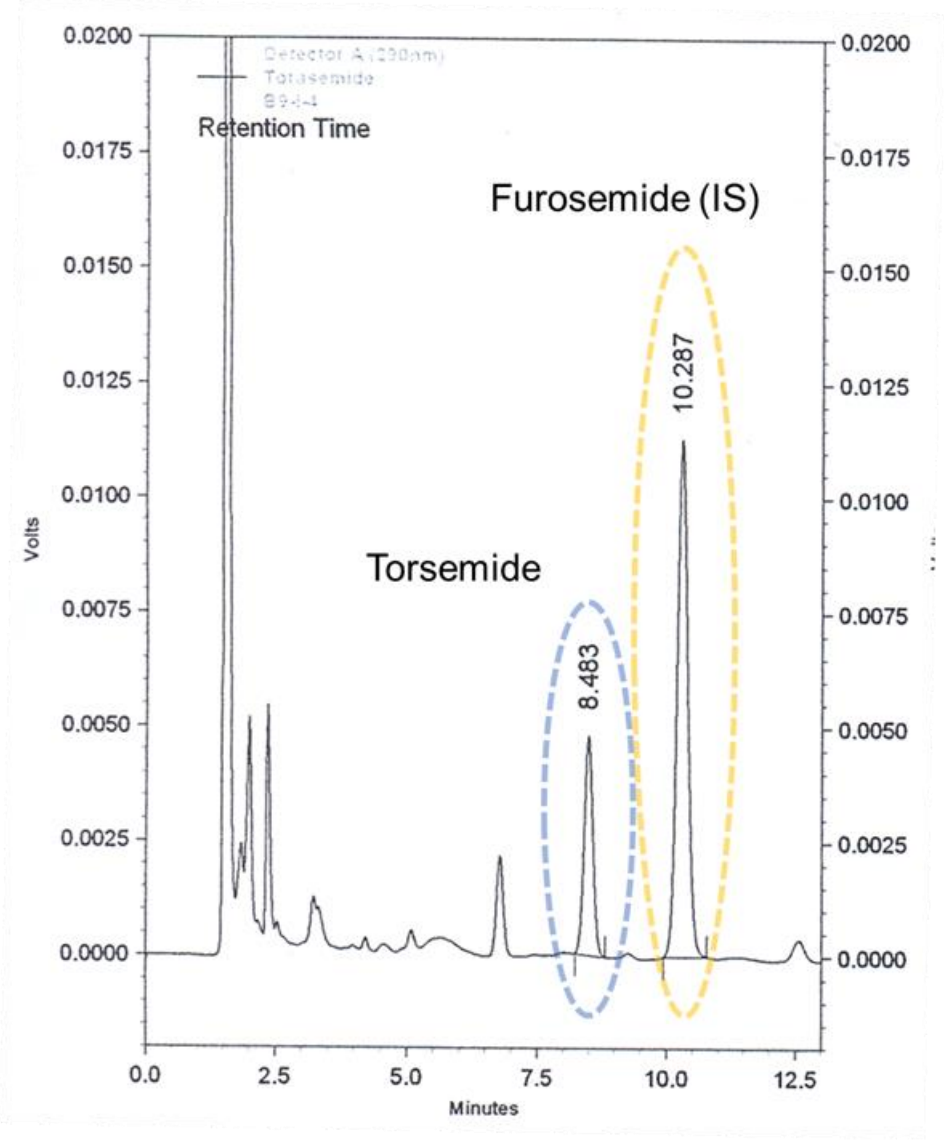

Figure S2. Representative peak chromatograms of torsemide and IS from human serum samples.

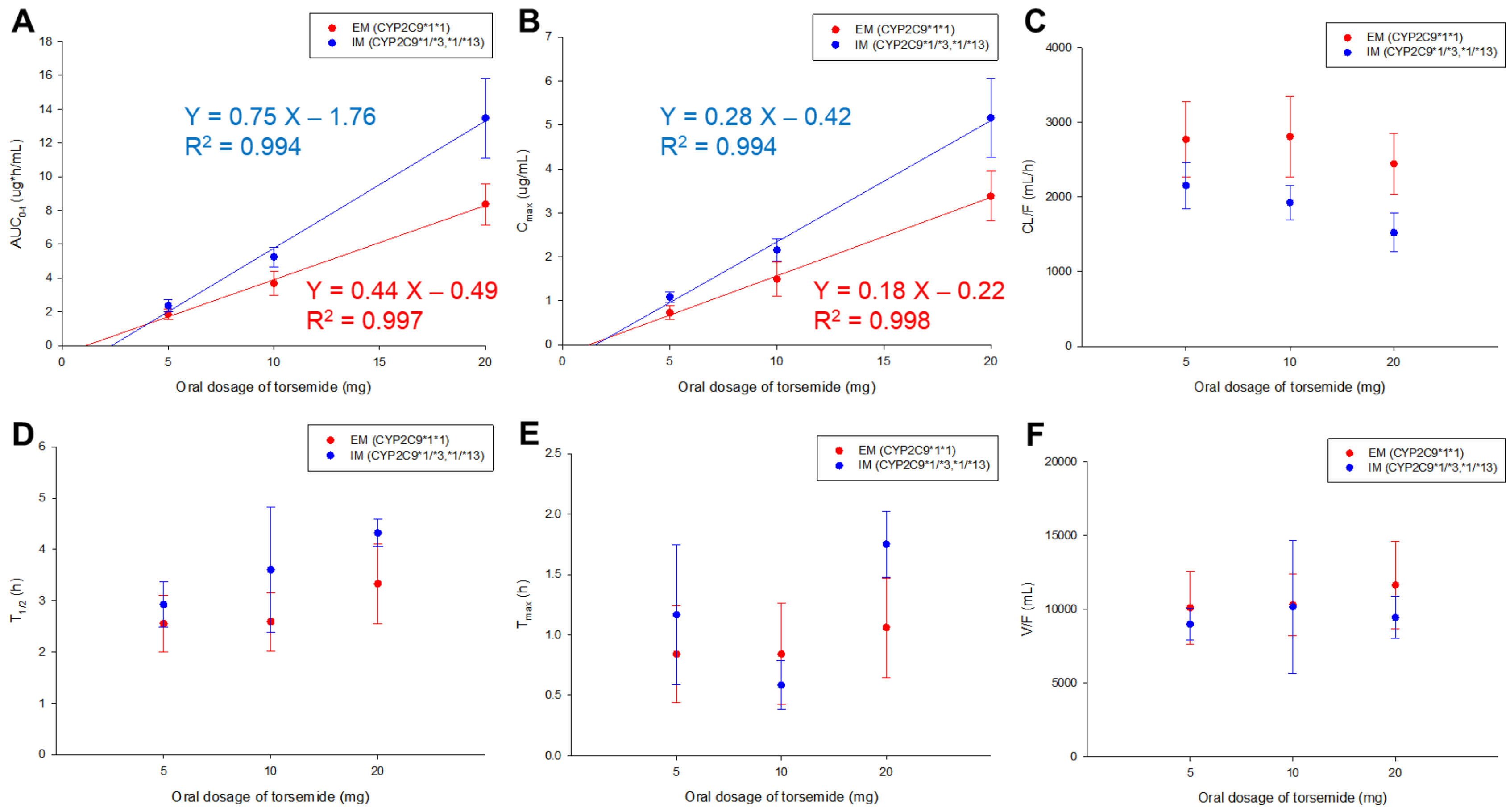

Figure S3. Graphs of changes in PK parameters [AUC0-t (A); Cmax (B); CL/F (C); T1/2 (D); Tmax (E); V/F (F)] by dose and CYP2C9 phenotype according to oral administration of torsemide in healthy Korean men. PK parameters were calculated by NCA. Vertical bar represents the SD at each point.

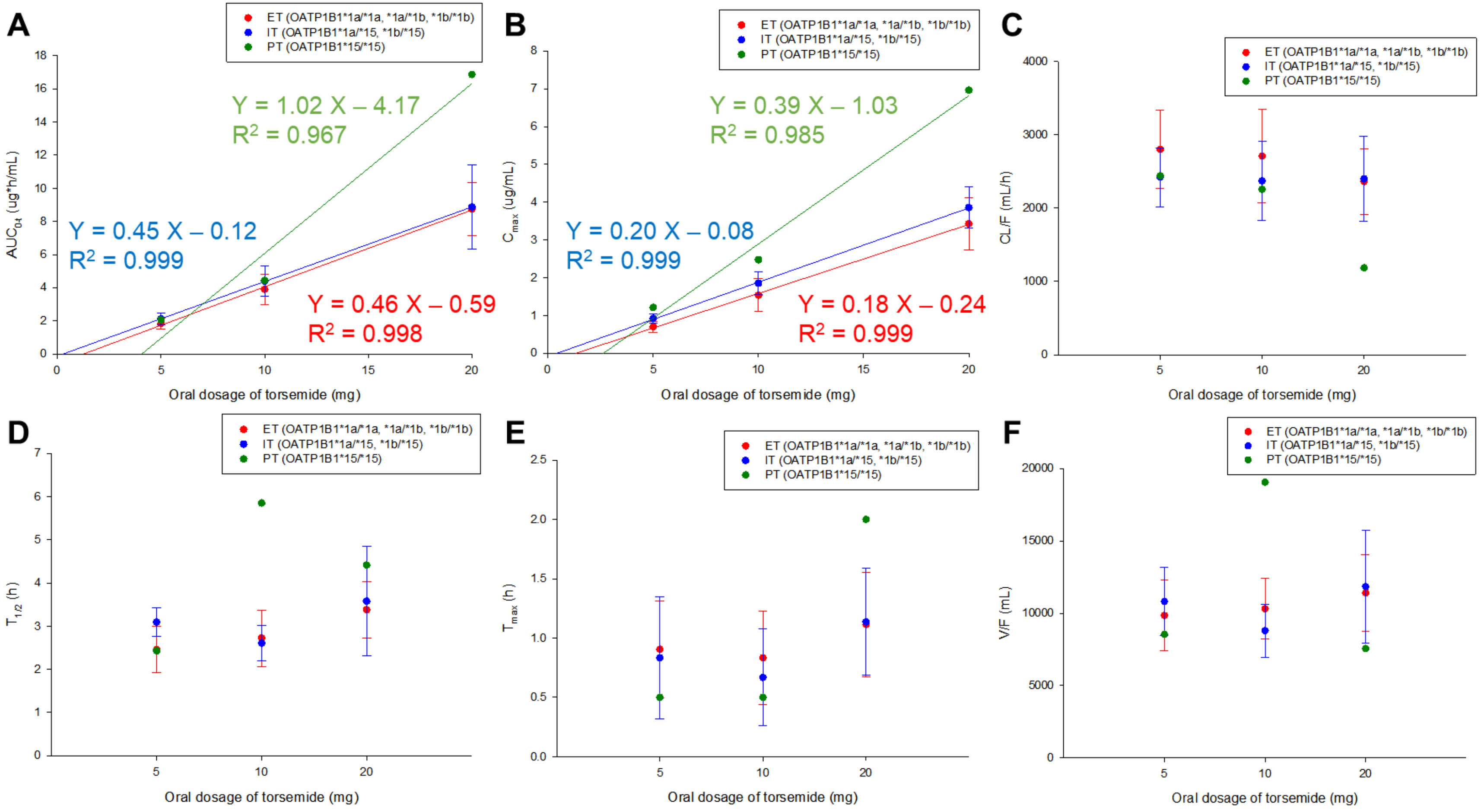

Figure S4. Graphs of changes in PK parameters [AUC0-t (A); Cmax (B); CL/F (C); T1/2 (D); Tmax (E); V/F (F)] by dose and OATP1B1 phenotype according to oral administration of torsemide in healthy Korean men. PK parameters were calculated by NCA. Vertical bar represents the SD at each point.

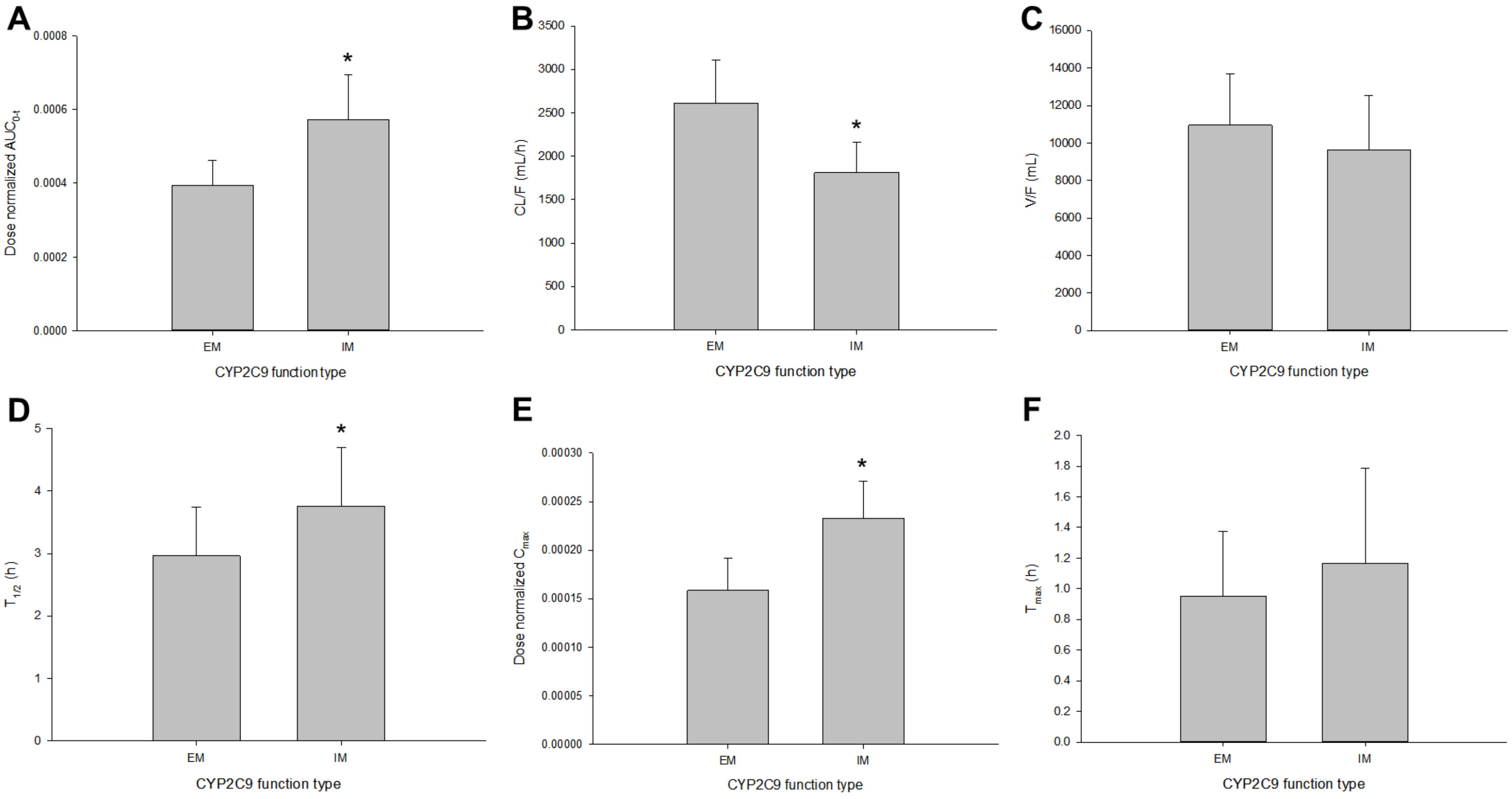

* means $p < 0.05$ (by *t* test).

Figure S5. Graphs of changes in PK parameters [Dose normalized AUC0-t (A); CL/F (B); V/F (C); T1/2 (D); Dose normalized Cmax (E); Tmax (F)] by CYP2C9 phenotype according to oral administration of torsemide in healthy Korean men. PK parameters were calculated by NCA. Vertical bar represents the SD.

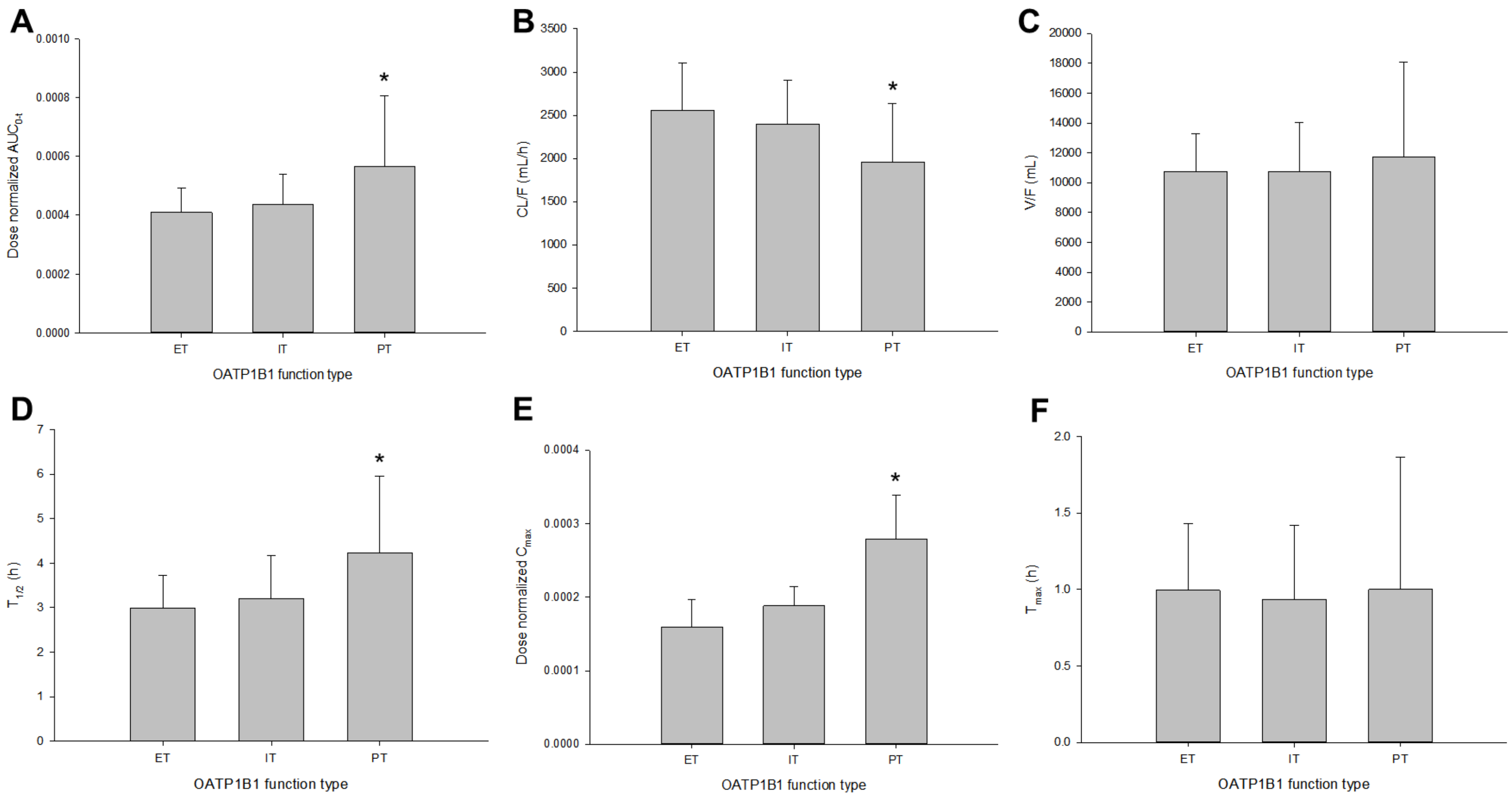

* means $p < 0.05$ (by Kruskal-Wallis one-way ANOVA test).

Figure S6. Graphs of changes in PK parameters [Dose normalized AUC0-t (A); CL/F (B); V/F (C); T1/2 (D); Dose normalized Cmax (E); Tmax (F)] by OATP1B1 phenotype according to oral administration of torsemide in healthy Korean men. PK parameters were calculated by NCA. Vertical bar represents the SD.

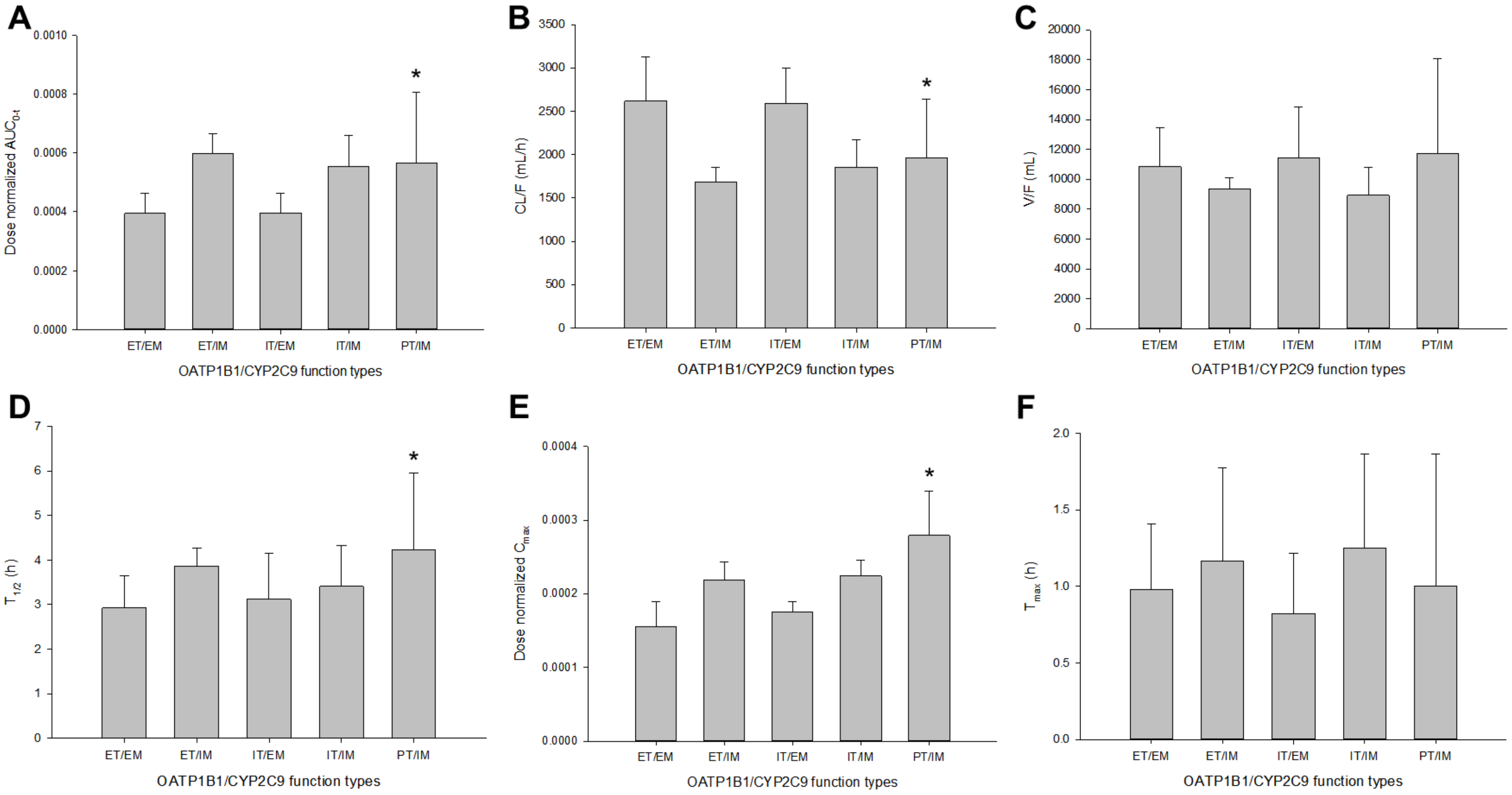

* means $p < 0.05$ (by Kruskal-Wallis one-way ANOVA test).

Figure S7. Graphs of changes in PK parameters [Dose normalized AUC0-t (A); CL/F (B); V/F (C); T1/2 (D); Dose normalized Cmax (E); Tmax (F)] by CYP2C9 and OATP1B1 phenotypes according to oral administration of torsemide in healthy Korean men. PK parameters were calculated by NCA. Vertical bar represents the SD.

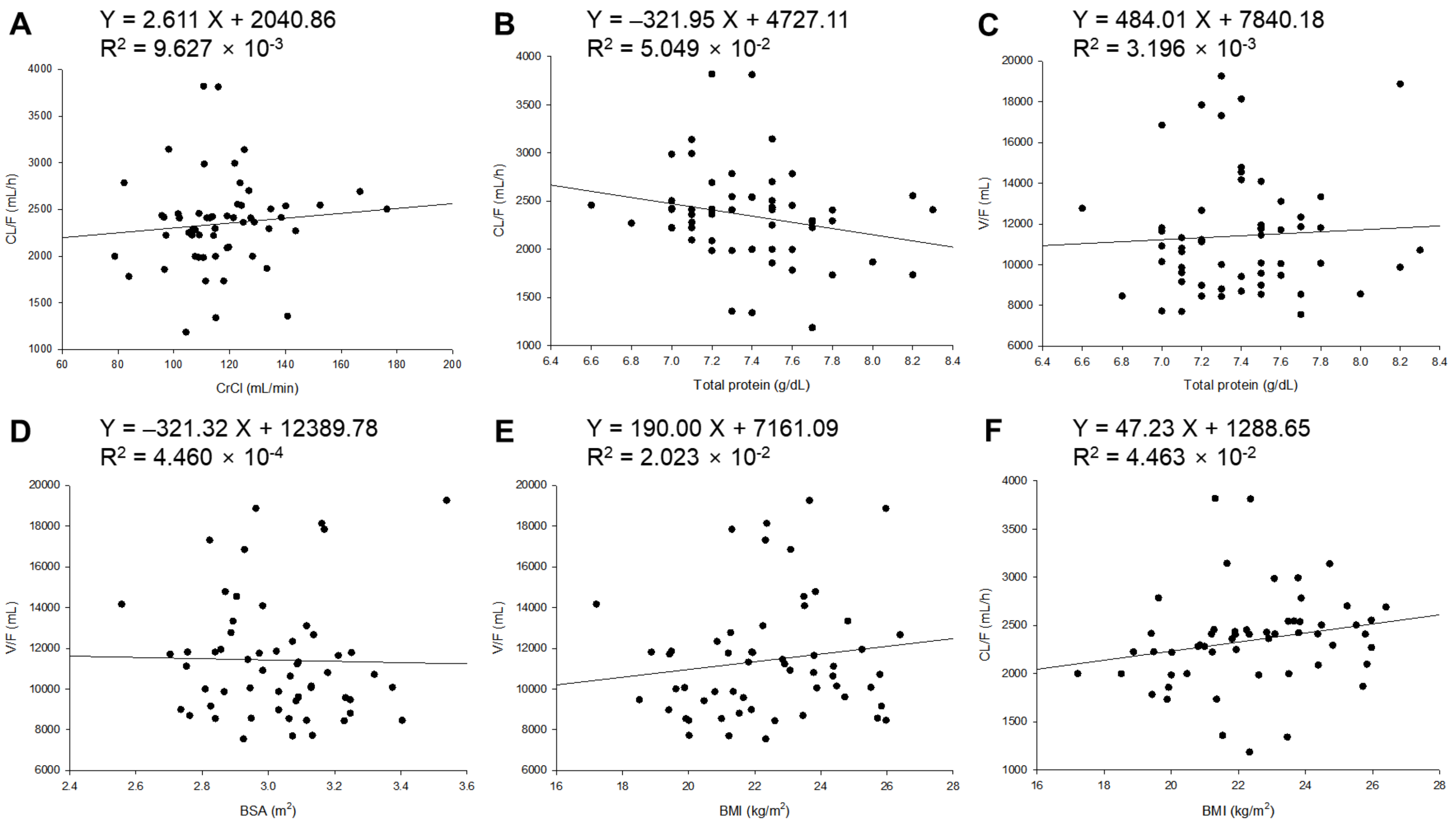

A
Y = 2.611 X + 2040.86
R2 = 9.627 × 10-3
CL/F (mL/h)
CrCl (mL/min)
B
Y = –321.95 X + 4727.11
R2 = 5.049 × 10-2
CL/F (mL/h)
Total protein (g/dL)
C
Y = 484.01 X + 7840.18
R2 = 3.196 × 10-3
V/F (mL)
Total protein (g/dL)
D
Y = –321.32 X + 12389.78
R2 = 4.460 × 10-4
V/F (mL)
BSA (m2)
E
Y = 190.00 X + 7161.09
R2 = 2.023 × 10-2
V/F (mL)
BMI (kg/m2)
F
Y = 47.23 X + 1288.65
R2 = 4.463 × 10-2
CL/F (mL/h)
BMI (kg/m2)

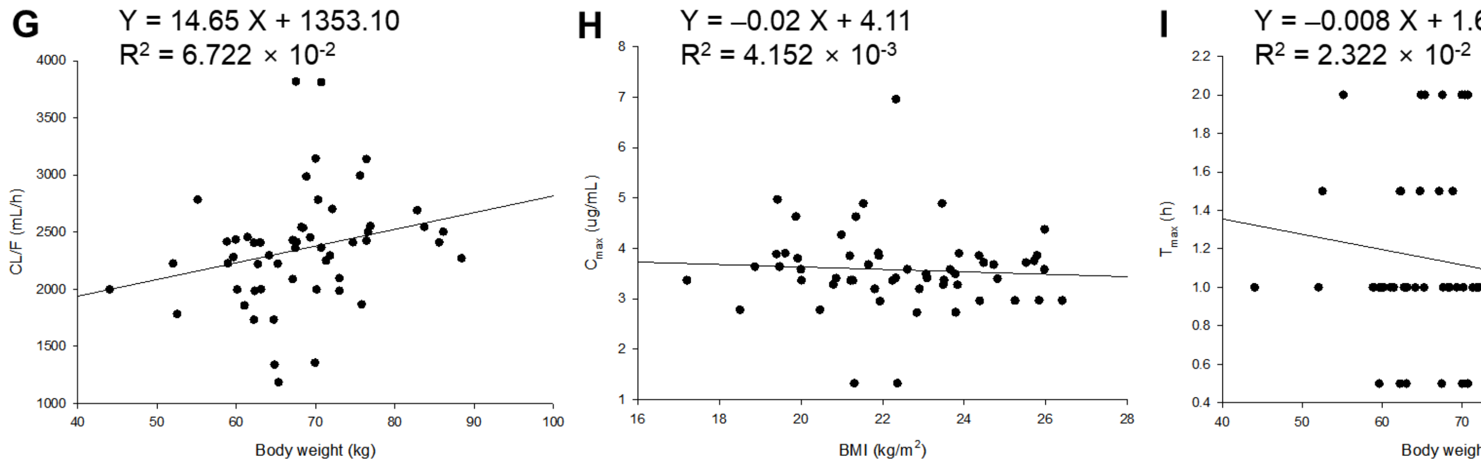

Figure S8. Correlation analysis graph between physiological or biochemical parameter values and PK parameter values [CrCl-CL/F (A); Total protein-CL/F (B); Total protein-V/F (C); BSA-V/F (D); BMI-V/F (E); BMI-CL/F (F); Body weight-CL/F (G); BMI-Cmax (H); Body weight-Tmax (I)] in healthy Korean males orally administered 20 mg torsemide (n = 56). PK parameters were calculated by NCA. CrCl was calculated based on the Cockcroft–Gault equation [(140-age) × body weight (kg)/serum creatinine (mg/dL) × 72] [11]. BMI was calculated based on the Kaup index [body weight (kg)/height2 (m2)] [12] (Hotta et al., 2005). BSA was calculated based on the Mosteller formula [√((height (cm) × weight (kg)/3600))] [13] (El Edelbi et al., 2012).

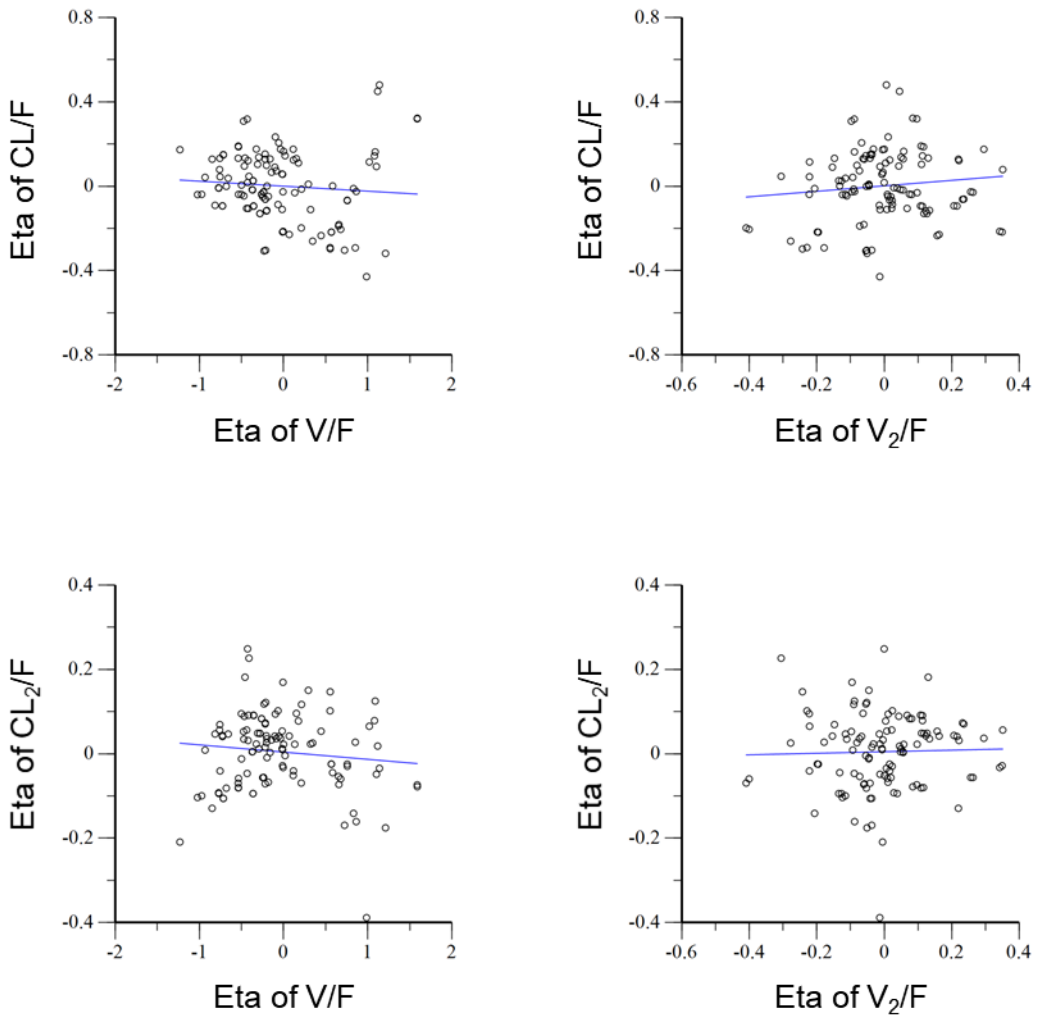

Figure S9. Eta plots between CL/F, CL2/F and V/F, V2/F in the final model for torsemide.

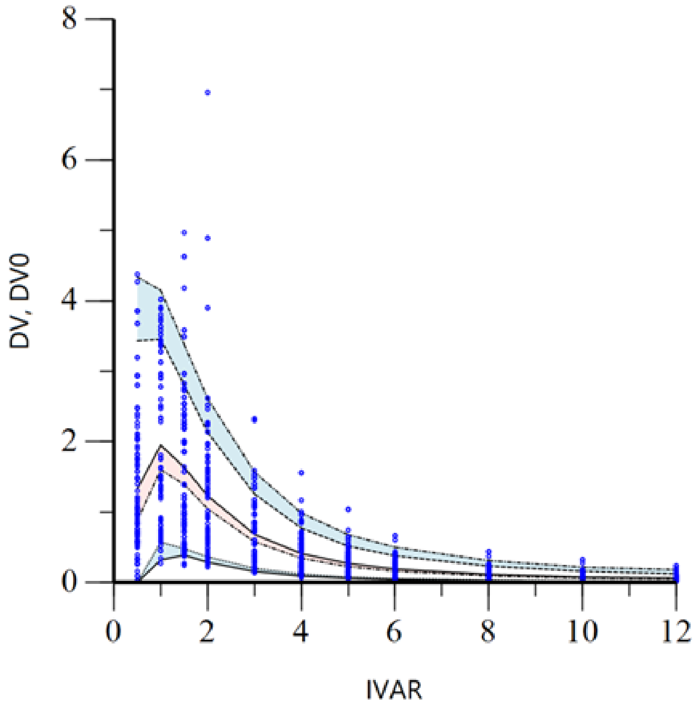

Figure S10. VPC of the final model for torsemide without any stratification. Observed concentrations were depicted by dots. The 95th, 50th, and 5th percentiles of the predicted concentrations are represented by black dashed lines. The 95% confidence intervals (CIs) for the predicted 5th and 95th percentiles are represented by blue shaded regions. The 95% CIs for the predicted 50th percentiles are represented by red shaded regions. IVAR (X-axis) means the time after oral torsemide administration. DV and DV0 (Y-axis) are observed concentrations of torsemide in the serum and concentrations predicted by the model, respectively.

1. Methods for determining genotypes

*1.1. Identification of CYP2C9*3 and *13 alleles*

Single nucleotide polymorphisms (SNP) of *CYP2C9*3* and **13* alleles were 1075A>C (I359L) and 269T>C (L90P), respectively. For genotyping of *CYP2C9*3* and **13* alleles, PCR was carried out in a 20 μL reaction mixture containing PCR PreMix, 200–300 ng of genomic DNA, and 10 pmol of each primer. Sequences of forward and reverse primers for CYP2C9*3 genotype were 5′-TGCACGAGGTCCAGAGGTAC-3′ and 5′-ACAAACTTAC-CTTGGGAATGAGA-3′. Sequences of forward and reverse primers for CYP2C9*13 genotype were 5′-TACAAATACAATGAAAATATCATG-3′ and 5′-CTAACAAC-CAGACTCATAATG-3′. PCR was conducted with an initial denaturation step (5 min and 94°C) followed by 30 cycles of denaturation (1 min and 94°C), annealing (1 min and 55°C), extension (1 min and 72°C), and final extension (10 min and 72°C). The amplified 105-bp fragment consisted of an intron of 10 bp and exon 7 of 95 bp. PCR products were visualized by ultraviolet (UV) transmission illumination after 2% agarose gel electrophoresis, staining with ethidium bromide. Restriction enzymes *KpnI* for *CYP2C9*3* and *Psp*GI for *13 were applied for digestion (for 1.5 h at 37°C) of PCR products. Digested fragments were visualized by UV after 2.5% agarose gel electrophoresis, staining with ethidium bromide. To confirm the reproducibility of PCR-RFLP, 5-10% of the total samples were directly sequenced using Real-Time PCR to perform a cross-platform confirmation

procedure. Dideoxy chain termination using BigDye technology (Applied Biosystems, Foster City, CA, USA) was applied for sequencing. Based on the $MgCl_2$/ethanol based protocol, PCR products were purified and performed on an ABI 3100 Genetic Analyzer (AB Applied Biosystems). The genotype agreement was 100% for all samples.

1.2. Identification of SLCO1B1 388A>G and 521T>C

SLCO1B1 388A>G was amplified with the following primers: 5′-ATAATGGTG-CAAATAAAGGGG-3′ (forward) and 5′-ACTATCTCAGGTGATGCTCTA-3′ (reverse). *SLCO1B1* 521T>C was amplified with the following primers: 5′-AAAGGAATCTGGGTCATACATGTGGATATACG-3′ (forward) and 5′-TTCAAAAGTAGACAAAGGGAAAGTGATCAT-3′ (reverse). A total volume of 20 μL of the reaction mixture was used to perform PCR and contained PCR PreMix, 200-300 ng genomic DNA, and 10 pmol of each primer. PCR was conducted with an initial denaturation step (5 min and 94°C) followed by 36 cycles of denaturation (30 sec and 94°C), annealing (30 sec and 57°C), extension (30 sec and 72°C), and final extension (6 min and 72°C). After PCR amplification, SNPs were detected by digestion with specific restriction enzymes (*Hinf* I for *SLCO1B1* 388A>G and *Mlu* I for *SLCO1B1* 521T>C). Restriction enzyme treatment conditions were as follows: *Hinf* I, 37°C for 3 h; and *Mlu* I, 37°C for 4 h. Digested PCR products were then separated by 3% agarose gel electrophoresis and visualized over an UV transilluminator after ethidium bromide staining. The 388A>G (N130D) SNP of the *SLCO1B1* gene corresponded to *1a and *1b. The 521T>C (V174A) SNP corresponded to *5. Simultaneous SNPs of 388A>G (N130D) and 521T>C (V174A) in the *SLCO1B1* gene corresponded to *15.

2. Assay conditions to determine serum torsemide concentration

2.1. Chromatographic conditions

Chromatographic separations were performed using a Shimadzu LC 10 AD*vp* System (Shimadzu Inc., Kyoto, Japan) consisting of the following parts: a degassing unit (DGU-12A), a high-pressure pump (LC-10AD*vp*), a column heating unit (CTO-10A*vp*), a UV detector (SPD-10A*vp*), and a system controller unit (SCL-10A*vp*). Shimadzu Model Class LC-10 system software (Shimadzu Inc.) was used for overall system operation and data processing. Separation of torsemide in serum samples was performed using a reversed-phase column, Shim-pack ODS (5 μm of particle size, 150 mm in length × 4.6 mm in inner diameter; Shimadzu Inc.). Ammonium acetate buffer (5 mM) containing 0.1% (v/v) acetic acid and acetonitrile were selected as mobile phases. The optimized ratio of acetate buffer to acetonitrile was 75:25 (v/v). The flow rate of the mobile phase was maintained at 1.0 mL/min and the total running time per sample was 13 min. The temperature of the column was maintained at 30°C using a heating unit. Torsemide separated from the reversed-phase column was quantified using a UV detector at a wavelength of 290 nm.

2.2. Calibration curve

Torsemide standard was dissolved in methanol to a concentration of 1 mg/mL. Furosemide was selected as an internal standard (IS). It was also dissolved in methanol to obtain a concentration of 40 μg/mL. Furosemide is a diuretic drug belonging to the same sulfonamide class as torsemide. It is structurally similar to torsemide. Figure S1 shows chemical structures of torsemide, an analyte, and furosemide, an IS. The torsemide standard solution was diluted step-by-step with methanol and then added to human blank serum (derived from at least six individuals) to prepare standard serum samples for a calibration curve with a final torsemide concentration of 0.02-10 μg/mL (0.02, 0.05, 0.1, 0.5, 1, 2, 5, and 10 μg/mL). To 0.5 mL of standard serum sample at each concentration, 50 μL of IS solution (furosemide of 40 μg/mL) and 50 μL of 1 M hydrochloric acid were added. After that, the mixture was vortex-mixed for 3 sec. After 5 mL of ethyl acetate was additionally added to the mixture, it was vortex-mixed for 1 min. The mixture was then centrifuged at 3,000 x *g* for 10 min. The organic layer, the supernatant, was then taken and transferred to a new tube. After it was evaporated and dried for about 1 h under a pure

nitrogen stream, the residue was reconstituted with 400 μL of mobile phase and centrifuged at 3,000 x *g* for 10 min. Then, using a Rheodyne injector (Shimadzu Inc.), 50 μL of the supernatant was injected into the HPLC system. A calibration curve was drawn for the theoretical torsemide concentration (X-axis) and the peak area ratio of torsemide to IS (Y-axis). During the analysis period, the calibration curve was drawn more than five times daily.

2.3. Sample preparation

Serum samples stored at -80°C after sampling from torsemide trial participants were thawed at 25°C prior to analysis and agitated for 3 sec. Pretreatment for serum samples was the same as the procedure used for preparing the calibration curve. Briefly, 0.5 mL of serum sample was mixed with 50 μL of 40 μg/mL furosemide IS solution, 50 μL of 1 M hydrochloric acid, and 5 mL of ethyl acetate, and the organic layer was dried, reconstituted, and injected into the HPLC-UV system. The sample injection volume into the analysis system was 50 μL. The concentration of torsemide in the serum sample (as the X-axis value of the calibration curve) was calculated by substituting the ratio of the peak area of torsemide to the peak area of the internal standard to the Y-axis of the calibration curve prepared on the day of analysis.

References

1. Lesne, M. Comparison of the pharmacokinetics and pharmacodynamics of torasemide and furosemide in healthy volunteers. *Arzneimittelforschung* **1988**, *38*, 160-163.
2. Cho, H.-Y.; Kang, H.-A.; Park, C.-H.; Kim, S.-M.; Kim, D.-H.; Park, S.; Kim, K.-R.; Hur, H.; Lee, Y.-B. Bioequivalence of Boryung torsemide tablet to Torem tablet (torasemide 10 mg) by high performance liquid chromatography/UV detector. *J Pharm Investig* **2005**, *35*, 323-328.
3. Kang, H.-A.; Yoon, H.; Lee, Y.-B. Bioequivalence of Torad tablet 5 mg to Torem tablet 5 mg (torasemide 5 mg). *J Pharm Investig* **2013**, *43*, 153-159.
4. Vargo, D.L.; Kramer, W.G.; Black, P.K.; Smith, W.B.; Serpas, T.; Brater, D.C. Bioavailability, pharmacokinetics, and pharmacodynamics of torsemide and furosemide in patients with congestive heart failure. *Clin Pharm Therap* **1995**, *57*, 601-609.
5. Lameire, N.; Dodion, L. Acute and chronic effects of torasemide in healthy volunteers. *Arzneimittelforschung* **1988**, *38*, 167-171.
6. Neugebauer, G.; Besenfelder, E.v.; Von Moellendorff, E. Pharmacokinetics and metabolism of torasemide in man. *Arzneimittelforschung* **1988**, *38*, 164-166.
7. Barr, W.; Smith, H.; Karnes, H.; Sica, D.; Vetticaden, S.; Purich, E.; Prasad, V.; Schary, W.; Kramer, W.; Linberg, S. Comparison of bioavailability, pharmacokinetics and pharmacodynamics of torasemide in young and elderly healthy volunteers. *Prog Pharmacol Clin Pharmacol* **1990**, *8*, 15-28.
8. Barr, W.; Smith, H.; Karnes, H.; Sica, D.; Vetticaden, S.; Prasad, V.; Kramer, W.; Scott, D.; Linberg, S. Torasemide dose-proportionality of pharmacokinetics and pharmacodynamics. *Prog Pharmacol Clin Pharmacol* **1990**, *8*, 29-37.
9. Kramer, W.G. Effect of Food on the Pharmacokinetics and Pharmacodynamics of Torsemide. *Am J Ther* **1995**, *2*, 499-503.

10. Kramer, W. Lack of effect of cimetidine on torsemide pharmacokinetics and pharmacodynamics in healthy subjects. In *Diuretics IV: chemistry, pharmacology and clinical applications*, Elsevier Science Publishers: 1993; pp. 361-364.
11. Hermsen, ED; Maiefski, MM; Florescu, MC; Qiu, F; Rupp, ME. Comparison of the modification of diet in renal disease and Cockcroft-Gault equations for dosing antimicrobials. *Pharmacother J Hum Pharmacol Drug Ther* **2009**, *29*, 649-655.
12. Hotta, M; Li, Y; Anme, T; Ushijima, H. Risk factors for low Kaup index among children in rural ethnic minority areas of Yunnan, China. *Pediatr Int* **2005**, *47*, 147-153.
13. El, Edelbi R; Lindemalm, S; Eksborg, S. Estimation of body surface area in various childhood ages–validation of the Mosteller formula. *Acta Paediatr* **2012**, *101*, 540-544.
